# “Living” Polymerization of Ethylene and 1-Hexene Using Novel Binuclear Pd–Diimine Catalysts

**DOI:** 10.3390/polym9070282

**Published:** 2017-07-15

**Authors:** Jianding Ye, Zhibin Ye

**Affiliations:** Bharti School of Engineering, Laurentian University, Sudbury, ON P3E 2C6, Canada

**Keywords:** ethylene polymerization, living polymerization, polyolefin catalysts, Pd–diimine, binuclear catalysts

## Abstract

We report the synthesis of two novel binuclear Pd–diimine catalysts and their unique behaviors in initiating “living” polymerization of ethylene and 1-hexene. These two binuclear catalysts, [(N^N)Pd(CH_2_)_3_C(O)O(CH_2_)*_m_*O(O)C(CH_2_)_3_Pd(N^N)](SbF_6_)_2_ (**3a**: *m* = 4, **3b**: *m* = 6) (N^N≡ArN=C(Me)–(Me)C=NAr, Ar≡2,6–(*i*Pr)_2_C_6_H_3_), were synthesized by simply reacting [(N^N)Pd(CH_3_)(N≡CMe)]SbF_6_ (**1**) with diacrylates, 1,4-butanediol diacrylate and 1,6-hexanediol diacrylate, respectively. Their unique binuclear structure with two identical Pd–diimine acrylate chelates covalently linked together through an ester linkage was confirmed by NMR and single crystal XRD measurements. Ethylene “living” polymerizations were carried out at 5 °C and under ethylene pressure of 400 and 100 psi, respectively, with the binuclear catalysts, along with a mononuclear chelate catalyst, [(N^N)Pd(CH_2_)_3_C(O)OMe]SbF_6_ (**2**), for comparison. All the polyethylenes produced with both binuclear catalysts show bimodal molecular weight distribution with the number-average molecular weight of the higher molecular weight portion being approximately twice that of the lower molecular weight portion. The results demonstrate the presence of monofunctional chain growing species resembling catalyst **2**, in addition to the expected bifunctional species leading to bifunctional “living” polymerization, in the polymerization systems. Both types of chain growing species exhibit “living” characteristics under the studied conditions, leading to the simultaneous linear increase of molecular weight in both portions. However, when applied for the “living” polymerization of 1-hexene, the binuclear catalyst **3a** leads to polymers with only monomodal molecular weight distribution, indicating the sole presence of monofunctional chain growing species. These two binuclear catalysts are the first Pd–diimine catalysts capable of initiating bifunctional ethylene “living” polymerization.

## 1. Introduction

“Living” polymerization is featured with chain growth in the absence of irreversible chain transfer and chain termination. “Living” polymerization techniques allow the precise control of polymer molecular weight and enable the tailored synthesis of polymers of well-defined chain architectures, such as block copolymers, star-shaped polymers, telechelic polymers, etc. The past decade has witnessed the tremendous developments in various “living” polymerization techniques, including radical [1,2,3], anionic [4,5], coordination [6,7,8], ring-opening metathesis polymerizations [9], etc. With respect to the “living” coordination polymerization of olefins, a number of well-behaved transition-metal catalyst systems have been successfully developed. Recent review papers [6,7,8] summarize the developments in the area of “living” olefin polymerization.

Bifunctional/multifunctional “living” polymerizations have received special research interest for the synthesis of polymers of designed complex architectures, such as telechelic polymers, block copolymers, star polymers, and graft polymers. Unlike the conventional monofunctional “living” polymerizations with one growing site per polymer chain, bifunctional/multifunctional “living” polymerizations are featured with the simultaneous chain growth from two/multiple identical active sites bound on a single polymer chain. With this outstanding feature, bifunctional “living” polymerization enables the synthesis of telechelic polymers capped with identical functionalities at both chain ends and block copolymers of symmetrical structure, for example, tri-block B–A–B copolymers with two identical B blocks using a simple two-step sequential addition of two different monomers. Multifunctional “living” polymerization instead facilitates the synthesis of star/graft polymers containing multiple arms/side branches. The design of bifunctional/multifunctional chain-initiating species is key to a successful bifunctional/multifunctional “living” polymerization system. In atom-transfer radical polymerization (ATRP), a number of bifunctional/multifunctional initiators containing two/multiple identical active halide groups have been successfully designed [2]. However, the development for binuclear/multinuclear transition metal catalysts for bifunctional/multifunctional olefin “living” polymerization still remains in the early stage, with only very few enabling binuclear/multinuclear catalyst systems reported in the literature. Murata et al. [10] reported a binuclear vanadium catalyst system for bifunctional/directional “living” polymerization of propylene, where the binuclear catalyst was in situ generated by reacting the V(acac)_3_ (acac = acetylacetonato)/AlEt_3_Cl catalyst system with an α,ω-nonconjugated diene. However, the binuclear catalyst in this system is not isolable. Yasuda et al. [11] reported binuclear lanthanide complexes for the preparation of polyolefin block copolymers by bifunctional polymerization. However, the polymers synthesized with the lanthanide complexes usually possess broad molecular weight distribution (with polydispersity index (*PDI*) often greater than 1.5) [7]. Bazan et al. [12] reported the synthesis of two binuclear Ni–α-iminocarboxamidato complexes capable of facilitating bifunctional “living” ethylene homo-and co-polymerizations.

**Scheme 1:**
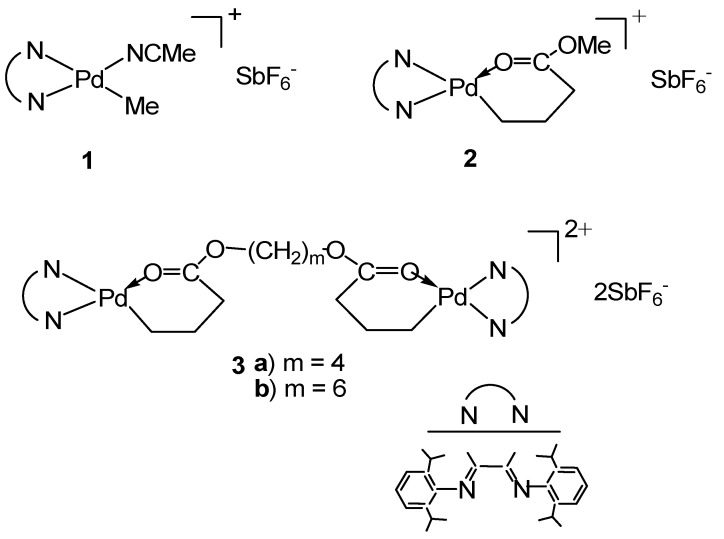
Representative mononuclear Pd–diimine catalysts (**1** and **2**) reported in the literature and binuclear catalysts (**3a**, **3b**) synthesized herein.

The discovery of Pd–diimine catalysts by Brookhart et al. in 1995 represents a major breakthrough in the area of “living” olefin polymerization [13,14,15,16,17,18,19,20,21,22,23,24,25,26,27,28,29,30]. This series of catalysts (see catalysts **1** and **2** in Scheme 1 for two representative ones) possesses three remarkable features, including capability of catalyzing “living” olefin polymerization [14,15,16,17,18,19,20,21,22,23,24,25,26,27,28,29,30], chain walking characteristics [13,31,32,33,34], and high tolerance of functional groups [35,36]. Typically, Pd–diimine catalysts have been reported to successfully facilitate the “living” polymerization of both ethylene and α-olefins at temperatures of ~5 °C [14,15,16,17,18,19,20,21,22,23,24,25,26,27,28,29,30]. Owing to their characteristic chain walking mechanism, Pd–diimine catalysts allow the novel synthesis of branched polyethylenes with controlled chain topologies in ethylene polymerization [31,32,33,34,37,38,39,40,41,42,43,44,45,46,47,48] and chain straightened poly(α-olefin)s with reduced branching density in α-olefin polymerization [14,16,49]. By employing their combined features, our group has tailor designed a family of polyethylenes of complex chain architectures, including hyperbranched, hybrid hyperbranched-linear, block, gradient and block-gradient, star, and surface-tethered polymer brushes, by Pd–diimine-catalyzed ethylene “living” polymerization. In particular, we have synthesized tri- and multinuclear Pd–diimine catalysts with 3 or multiple metal centers tethered uniquely onto the common small well-defined or large polymer core through their initiating sites, which enabled the first synthesis of 3- or multiarm star polyethylenes by tri- or multifunctional ethylene “living” polymerization [21,22,28]. However, binuclear Pd–diimine catalysts facilitating bifunctional ethylene “living” polymerization have not been reported to date. Recently, several binuclear Pd–diimine catalysts have been reported for ethylene polymerization and copolymerization [50,51,52]. Therein, the binuclear metal centers are bound together through their diimine spectator ligands and thus cannot facilitate bifunctional ethylene polymerization though showing significant cooperative binuclear effect relative to mononuclear analog.

Further to our earlier works, we report in this paper the synthesis of two novel binuclear Pd–diimine catalysts, [(N^N)Pd(CH_2_)_3_C(O)O–(CH_2_)*_m_*–O(O)C(CH_2_)_3_Pd(N^N)](SbF_6_)_2_ (**3a**: *m* = 4, **3b**: *m* = 6; see Scheme 1) (N^N≡ArN=C(Me)–(Me)C=NAr, Ar≡2,6–(*i*Pr)_2_C_6_H_3_), and their performances in catalyzing/initiating the “living” polymerization of ethylene and 1-hexene. With the two metal centers covalently joined together via their chain initiating sites, we demonstrate that these binuclear catalysts can facilitate the bifunctional “living” polymerization of ethylene. To the best of our knowledge, these are the first binuclear Pd–diimine catalysts having the capability of initiating the bifunctional ethylene “living” polymerization.

## 2. Materials and Methods

### 2.1. Materials

All manipulations involving air- and/or moisture-sensitive compounds were carried out in a N_2_ filled drybox or using Schlenk techniques. Ultra-high purity N_2_ and polymer-grade ethylene (both from Praxair, Sudbury, Canada) were purified by passing through 3 Å/5 Å molecular sieve and Oxiclear columns to remove moisture and oxygen, respectively, before use. Chlorobenzene (99.5%, Aldrich, Oakville, Canada) was refluxed over CaH_2_ and distilled before use. Methyl acrylate (99%), 1,4-butanediol diacrylate (technical grade, 90%), and 1,6-hexanediol acrylate (technical grade, 80%) were purchased from Aldrich, dried over 4 Å molecule sieves, and degassed with N_2_. 1-Hexene (99%, Aldrich) was dried over sodium, and distilled under N_2_ before storing over 4 Å molecular sieves. The diimine ligand, ArN=C(Me)–(Me)C=NAr (Ar≡2,6–(*i*Pr)_2_C_6_H_3_), the acetonitrile adduct, [(ArN=C(Me)–(Me)C=NAr)Pd(CH_3_)(N≡CMe)]SbF_6_ (**1**), and the mononuclear chelate catalyst [(ArN=C(Me)–(Me)C=NAr)Pd(CH_2_)_3_C(O)OMe] SbF_6_ (**2**) were synthesized according to literature procedures [9]. Other chemicals, including CH_2_Cl_2_ (anhydrous), diethyl ether (anhydrous), *n*-pentane (anhydrous), triethylsilane (97%), etc., were purchased from Aldrich and used as received.

### 2.2. Measurements

Nuclear magnetic resonance (NMR) spectra were recorded on a Varian Gemini 2000 (Palo Alto, CA, USA) or a Bruker AV500 spectrometer (Karlsruhe, Germany) at ambient temperature. CD_2_Cl_2_ was used as the solvent for the organometallic compounds and CDCl_3_ was used as the solvent for the polymer samples. Gel permeation chromatography (GPC) elution curves of the polymer samples were measured on a Waters Alliance 2965 (Milford, MA, USA), Separation Module equipped with a Waters 2410 differential refractive detector and three Polymer Laboratory 30 cm mixed columns (PLgel 10 μm MIXED-B 300 × 7.5 mm). The system operated at 30 °C, with THF as the eluent at a flow rate of 1.0 mL/min. Narrowly distributed polystyrene standards with molecular weights from 580 to 6,035,000 g/mol were used for the relative column calibration. Single crystal X-ray diffraction (XRD) was performed on a Bruker SMART APEX2 Mo diffractometer at −100 °C. See the earlier papers [18,21] from our group for details on the characterization.

### 2.3. Synthesis of [(N^N)Pd(CH_2_)_3_C(O)O–(CH_2_)_4_–O(O)C(CH_2_)_3_Pd(N^N)](SbF_6_)_2_ (**3a**; N^N≡ArN=C(Me)–(Me)C=NAr; Ar≡2,6–(iPr)_2_C_6_H_3_)

A Schlenk flask was charged with the acetonitrile complex **1** (0.5 g, 6.22 × 10^−4^ mol). Diethyl ether (25 mL) was added, followed by the addition of 0.0616 g (3.11 × 10^−4^ mol) of 1,4-butanediol diacrylate and 25 mL of CH_2_Cl_2_. The bright yellow solution was stirred under nitrogen for 2 days at room temperature. The resulting solution was filtered, and the solvent was removed in vacuo. The resulting solids were redissolved in 10.0 mL of CH_2_Cl_2_. Subsequently, 20 mL of pentane was added slowly to yield an orange precipitate and the supernatant was decanted carefully. This dissolution-precipitation procedure was repeated for several times. The final precipitate was washed with 20 mL of pentane twice and then dried in vacuo to yield 0.25 g of orange powder of **3a** (46.7% yield).

^1^H NMR (500 MHz, CD_2_Cl_2_, room temperature): δ (ppm) 7.40–7.26 (m, 12, H_aryl_), 3.24 (t, 4, OCH_2_CH*_2_*), 2.98 (septet, 4, CHMe_2_), 2.96 (septet, 4, C′′HMe_2_), 2.42 (t, 4, CH_2_C(O)), 2.24 and 2.23 (s, 3 each, N=C(Me)–C′(Me)=N), 1.41 (t, 4, PdCH_2_), 1.38, 1.37, 1.32, 1.30, 1.29, 1.26, 1.23 and 1.22 (s, 6 each, CHMeMe′, C′HMeMe′), 1.25 (m, 4, OCH_2_CH_2_), 0.67 (pentet, 4, PdCH_2_CH_2_CH_2_C(O)).

^13^C NMR (125 MHz, CD_2_Cl_2_, room temperature): δ (ppm) 183.0 [PdCH_2_CH_2_CH_2_C(O)], 179.4 and 172.3 (N=C–C′=N), 141.0 and 140.9 (Ar, Ar′, C_ipso_), 138.9 and 138.4 (Ar, Ar′, C_o_), 129.2 and 128.1 (Ar, Ar′ C_p_), 125.0 and 124.4 (Ar, Ar′ C_m_), 69.0 (C(O)OCH_2_CH_2_), 36.2 and 30.3 (PdCH_2_CH_2_CH_2_C(O)), 29.6 and 29.3 (CHMe_2_, C′HMe_2_), 28.3 (C(O)OCH_2_CH_2_), 23.9 (PdCH_2_CH_2_CH_2_C(O)), 24.2, 24.1 23.5 and 23.4 (CHMeMe′, C′HMeMe′), 21.8 and 20.1 (N=C(Me)–C′(Me)=N).

The 5-member chelate isomer resulting from 1,2-acrylate insertion was found (13%) from the NMR spectra. ^1^H NMR (CD_2_Cl_2_, 500 MHz, room temperature): δ (ppm) 3.80 (t, 2, *J* = 6.78, OCH_2_CH_2_), 2.53 (m, 1, CHMeC(O)), 2.24 and 2.22 (s, 3 each, N=C(Me)–C′(Me)=N), 1.03 (d, 3, *J* = 7.09, CHMeC(O)). ^13^C NMR (CD_2_Cl_2_, 125 MHz, room temperature δ): 194.2 (C(O)), 178.9 and 173.0 (N=C–C′=N), 68.9 (OCH_2_CH_2_), 44.6 (CHMeC(O)), 29.0 (PdCH_2_), 28.2 (OCH_2_*C*H_2_), 21.4 and 19.8 (N=C(Me)–C′(Me)=N), 18.4 (CHMeC(O)).

### 2.4. Synthesis of [(N^N)Pd(CH_2_)_3_C(O)O–(CH_2_)_6_–O(O)C(CH_2_)_3_Pd(N^N)](SbF_6_)_2_ (**3b**; N^N≡ArN=C(Me)–(Me)C=NAr; Ar≡2,6–(iPr)_2_C_6_H_3_)

This compound was synthesized in the same procedure as **3a** except that 1,6-hexanediol diacrylate was used. After three cycles of dissolution-precipitation procedure using dichloromethane and pentane, the final precipitate was dried in vacuo to give 0.23 g of orange powder of **3b** (42.2% yield). Anal. Calcd (found) for C_70_H_104_F_12_N_4_O_4_Pd_2_Sb_2_: C, 48.04 (48.36); H, 5.99 (5.82); N, 3.20 (3.36). ESI MS *m*/*z* calculated (found): [C_70_H_104_N_4_O_4_Pd_2_]^2+^, 639.3 (639.2); [C_70_H_104_F_6_N_4_O_4_Pd_2_Sb]^+^, 1513.5 (1513.6).

^1^H NMR (500 MHz, CD_2_Cl_2_, room temperature): δ (ppm) 7.40–7.26 (m, 12, H_aryl_), 3.24 (t, 4, *J* = 6.21, OCH*_2_*CH_2_CH_2_), 2.98 (septet, 4, *J* = 6.94, CHMe_2_), 2.96 (septet, 4, *J* = 6.94, C′′HMe_2_), 2.42 (t, 4, *J* = 5.68, CH*_2_*C(O)), 2.24 and 2.23 (N=C(Me)–C′(Me)=N), 1.41 (t, 4, *J* = 6.15, PdCH*_2_*), 1.38, 1.37, 1.32, 1.30, 1.29, 1.26, 1.23 and 1.22 (s, 6 each. *J* = 6.94 and 6.78, CHMeMe′, C′HMeMe′), 1.25 (m, 4, OCH_2_CH_2_CH_2_), 1.07 (pentet, *J* = 3.63, OCH_2_CH_2_CH_2_), 0.67 (pentet, 4, *J* = 5.68, PdCH_2_CH_2_CH_2_C(O)).

^13^C NMR (125 MHz, CD_2_Cl_2_, room temperature): δ (ppm) 183.0 [PdCH_2_CH_2_CH_2_C(O)], 179.4 and 172.4 (N=C–C′=N), 141.1 and 141.0 (Ar, Ar′, C_ipso_), 139.0 and 138.4 (Ar, Ar′, C_o_), 129.2 and 128.2 (Ar, Ar′ C_p_), 125.0 and 124.5 (Ar, Ar′ C_m_), 69.0 (C(O)OCH_2_), 36.2 and 30.3 (PdCH_2_CH_2_CH_2_C(O)), 29.6 and 29.3 (CHMe_2_,C′HMe_2_), 28.3 (C(O)OCH_2_CH_2_CH_2_), 25.7 (C(O)OCH_2_CH_2_CH_2_), 24.0 (PdCH_2_CH_2_CH_2_C(O)), 24.2, 24.1 23.5 and 23.4 (CHMeMe′, C′HMeMe′) 21.8 and 20.1 (N=C(Me)–C′(Me)=N).

The 5-member chelate isomer resulting 1,2-acrylate insertion was found (14%) based on the NMR spectra. ^1^H NMR (CD_2_Cl_2_, 500 MHz, room temperature): δ (ppm) 3.80 (t, 2, *J* = 6.78, OCH_2_CH_2_CH_2_), 2.53 (m, 1, CHMeC(O)), 2.24 and 2.22 (s, 3 each, N=C(Me)–C′(Me)=N), 1.03 (d, 3, *J* = 7.09, CHMeC(O)). ^13^C NMR (CD_2_Cl_2_, 125 MHz, room temperature): δ (ppm) 194.3 (C(O)), 178.8 and 173.1 (N=C–C′=N), 69.9 (OCH_2_CH_2_CH_2_), 44.7 (CHMeC(O)), 29.0 (PdCH_2_), 28.2 (OCH_2_CH_2_CH_2_), 25.6 (OCH_2_CH_2_*C*H_2_), 21.4 and 19.8 (N=C(Me)–C′(Me)=N), 18.5 (CHMeC(O)).

### 2.5. General Procedure for Ethylene “Living” Polymerization

Ethylene “living” polymerizations were performed in a 500 mL Autoclave Engineers Zipperclave reactor equipped with a MagneDrive agitator, a removable heating/cooling jacket, and a sampling port. The reactor temperature was maintained by the heating/cooling jacket. The reactor was washed with acetone, heated under vacuum at 80 °C, then cooled down to 5 °C. Chlorobenzene (280 mL) was then injected into the reactor under N_2_ protection. A freshly prepared catalyst (**3a** or **3b**) solution in chlorobenzene (20 mL; containing 0.05 mmol of catalyst) was subsequently injected into the reactor under N_2_ protection. After thermal equilibrium at 5 °C, the reactor was pressurized with ethylene to 400 psi to start the polymerization. During the polymerization, ethylene pressure was maintained constant at 400 psi by continuous feed from a cylinder and the temperature was maintained at 5 °C. Every 1 h, a 20 mL aliquot of the polymerization solution was taken from the reactor sampling port and quenched by addition of 0.1 mL of triethylsilane. Solvent was removed from each aliquot via evaporation, leaving a sticky brown black polymer residue. At the end of polymerization run (6 h), ethylene pressure was released and 0.2 mL of triethylsilane was added to quench the catalyst. The polymer solution was collected and the polymer was obtained by precipitation in a large amount of methanol. All the polymer samples were redissolved in THF or petroleum ether, filtered using a 0.2 μm syringe filter, and then precipitated in methanol. Finally, the polymer samples were dried in a vacuum oven at 50 °C for three days and weighed.

### 2.6. General Procedure for “Living” Polymerization of 1-Hexene

“Living” polymerization of 1-hexene was carried out in a 250 mL Schlenk flask reactor equipped with a magnetic stirrer. Catalyst **3a** (0.05 mmol) was weighed into a dried Schlenk flask in a N_2_-filled drybox. Ten milliliters of dichloromethane was added, dissolving the catalyst to form a light orange/yellow solution. 1-Hexene (15.0 mL) and dichloromethane (80 mL) were added under nitrogen to the dried polymerization reactor. The reactor was then placed in an ice bath and the solution was stirred for more than 30 min to establish the polymerization temperature. The catalyst solution was then injected into the reactor to start the polymerization. Every 30 min for 3 h, a 10 mL aliquot of the polymerization solution was removed and quenched by addition of 0.1 mL of triethylsilane. Solvent was removed from each aliquot by evaporation. The resulting polymer samples were redissolved in THF and the solutions were filtered using a 0.2 μm syringe filter. The polymers were obtained by precipitation in methanol and were dried in a vacuum oven at 50 °C for three days.

### 2.7. Polymer Cleavage by Hydrolysis

A typical procedure is as follows. A 0.05 g of polymer sample 3a-E400-6, the polymer obtained after 6 h of “living” ethylene polymerization using **3a** at 400 psi (bimodal GPC curve with *M_n_* = 77 kg/mol, *PDI* = 1.25), was dissolved in 15 mL of THF in a 50 mL round-bottomed flask equipped with a condenser and a N_2_ inlet. To this solution was added 1 mL of KOH solution (1 M solution in methanol), and the mixture was refluxed for 24 h. The solution was then evaporated to dryness and redissolved in THF. The solution was filtrated, and precipitated using methanol. The polymer was dried under vacuum to give 0.045 g cleaved sample (yield = 90%). GPC measurement of the cleaved polymer (THF vs. PS standard): *M_n_* = 54 kg/mol and *PDI* = 1.17. ^1^H NMR (CDCl_3_, 200 MHz, room temperature): The peak at 4.05 ppm (m, COOCH_2_CH_2_) disappeared after cleavage, and the peak at 2.27 ppm (t, CH_2_C(O)) shifted to 2.37 ppm after cleavage.

## 3. Results and Discussion

### 3.1. Synthesis of Binuclear Pd–Diimine Chelate Complexes

The acetonitrile adduct **1** and acrylate chelate complex **2** are commonly used Pd–diimine catalysts for “living” polymerization of ethylene and α-olefins [15,16]. The acrylate chelate complexes (such as **2** as a typical example) can be easily synthesized by reaction of **1** with various acrylate species (Equation (1)).


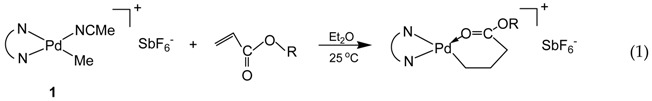


In this reaction, the acrylate vinyl bond is inserted into the Pd–Me bond of **1** via a 2,1-insertion mechanism followed by rearrangement (via β-hydride elimination and reinsertion) to form the six-membered chelates, which does not allow further insertion of acrylates [35,36]. In olefin “living” polymerization catalyzed with the Pd–diimine acrylate chelate complexes, chain propagation starts by monomer insertion into the Pd–CH_2_ bond and this yields uniquely polymer chains end-capped with a ester group (Equation (2)), which is introduced at the beginning of the chain [16].


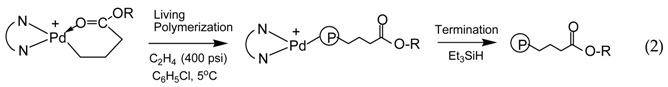


On the contrary, the polymers synthesized by “living” polymerization using **1** are fully saturated and unfunctionalized. With the unique synthesis and polymerization chemistry, our group has synthesized various acrylate chelate complexes [17,18,19,20,21,22,28], including chelate complexes containing functional groups [18,19,28], and trinuclear [21] and multinuclear Pd–diimine complexes [22], which facilitate the design of branched polyethylenes of various new chain architectures.

Taking advantage of the chemistry of the acrylate chelate, we synthesized in this work two binuclear Pd–diimine chelate complexes, **3a** and **3b**, by reacting **1** with two commercially available diacrylates, 1,4-butanediol diacrylate and 1,6-hexanediol diacrylate, respectively, using a 2:1 molar ratio between **1** and the diacrylates (Equation (3)).


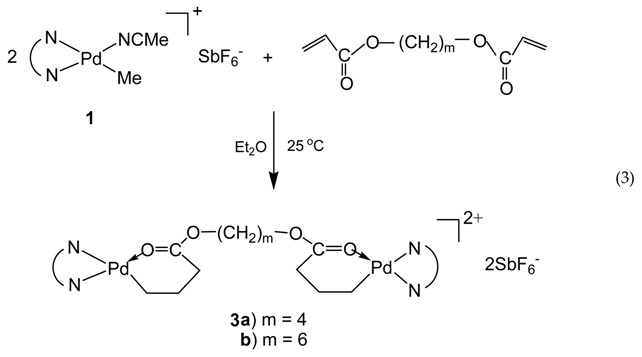


**Figure 1:**
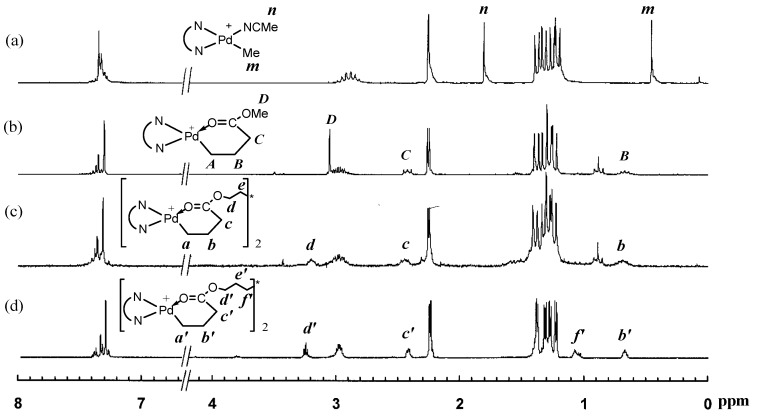
^1^H NMR spectra for the Pd–diimine compounds: (**a**) **1**; (**b**) **2**; (**c**) **3a**; and (**d**) **3b**. The resonance peaks from the diamine ligand are not labeled. Peaks for *A*, *a*, *e*, *a*’, and *e*’ overlap with the peaks for methyl protons (1.2–1.45 ppm) on the isopropyl groups of the diimine ligand.

In this reaction, both acrylate groups of the diacrylate monomers reacted with **1** leading to the binuclear complexes with two Pd–diimine acrylate chelates connected together through the ester linkage. The binuclear structure of the two complexes was confirmed by using ^1^H and ^13^C NMR spectroscopy. Figure 1 shows the ^1^H NMR spectra of the two binuclear complexes along with those of **1** and **2** for comparison. The resonance peaks at 0.46 ppm for PdMe and 1.81 ppm for PdNCMe in **1** (*m* and *n* in Figure 1a, respectively) are absent in the two binuclear complexes (Figure 1c,d)), showing the absence of unreacted **1** in **3a** and **3b**. Moreover, there are no peaks observed in the vinyl double bond region, which indicates the complete reaction of the acrylate groups in the diacrylates. The six-membered chelate structure in **3a** and **3b** is validated from the peaks at 2.42 ppm for CH_2_C(O) (*c* and *c*’ in Figure 1c,d, respectively) and 0.67 ppm for PdCH_2_CH_2_CH_2_C(O) (*b* and *b*’ in Figure 1c,d, respectively) [35].

**Scheme 2:**
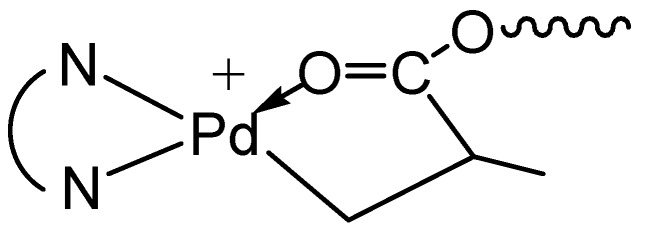
The five-membered chelate isomer.

As shown by Brookhart et al., four-membered or five-membered chelate isomers are often found in the acrylate chelated Pd–diimine complexes though the six-membered chelate is always predominant [35]. In the ^13^C NMR spectra of both binuclear complexes (see Figures S1 and S2 in Supplementary Materials), a resonance peak at 194.3 ppm for C(O), typically for the five-membered chelate isomer (Scheme 2) resulting from 1,2-insertion of the acrylate group into the Pd–Me of **1** followed by rearrangement [35], is found. For the six-membered chelate, the peak for C(O) locates at 183.0 ppm. Integration shows that the five-membered chelate takes a percentage of about 13% in both binuclear complexes. Other chelate isomers were not found.

**Figure 2:**
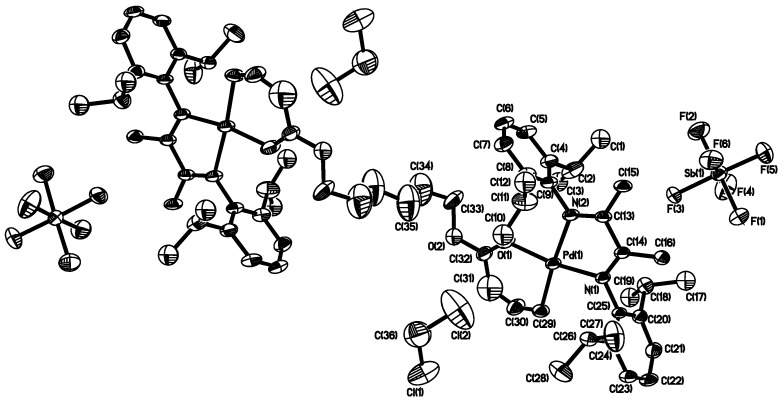
Thermal ellipsoid (30% probability) of **3b**. Hydrogen atoms and the disordered parts of the structure are omitted. Selected bond lengths (Å) and angles (°): Pd(1)–N(1) = 2.014(3), Pd(1)–N(2) = 2.131(3), Pd(1)–C(29) = 2.027(4), Pd(1)–O(1) = 2.052(3); N(1)–Pd(1)–N(2) = 77.60(13), N(1)–Pd(1)–C(29) = 97.07(17), C(29)–Pd(1)–O(1) = 91.76(17), O(1)–Pd(1)–N(2) = 93.59(14), N(1)–Pd(1)–O(1) = 171.03(13), C(29)–Pd(1)–N(2) = 174.56(17).

In particular, the binuclear structure and purity of complex **3b** were also confirmed by characterization using electrospray ionization mass spectrometry (see Figure S3 in Supplementary Materials) and elemental analysis, in addition to NMR spectra. The structure was further confirmed by using single crystal X-ray diffraction (XRD) analysis. Single crystals of **3b** were obtained by layering pentane and diethyl ether into a dichloromethane solution of **3b**. X-ray diffraction measurement was conducted to elucidate the molecular structure of **3b**. Figure 2 shows the thermal ellipsoid plot (30% probability) of the complex, which shows its binuclear structure with two Pd chelates joined together through the ester linkage (see Figures S4 and S5, and Tables S1–S6 in Supplementary Materials for detailed crystallographic and molecular structure data). Owing to the structural symmetry, atoms on half of the binuclear complex are labeled in the plot. Two dichloromethane molecules are incorporated in the crystal lattice. The SbF_6_^−^ anions are far apart and do not show any interactions with the metal centers. A perusal of the bond angles around the metal center of complexes shows that the coordination geometry around the palladium center is distorted square-planar. The sterically bulky isopropyl substituted aryl rings are nearly perpendicular to the plane of the butandiimino moiety with the dihedral angles of 85.95° and 84.22°, respectively. The five-membered chelate isomer was not found in the single crystal analyzed. All the evidences above confirm the binuclear structure present in both **3a** and **3b**, as well as their high purity with no residual mononuclear **1** or the singly chelated complex with an unreacted pendant acrylate group observed.

### 3.2. Ethylene “Living” Polymerization with **2**, **3a**, and **3b** at 400 psi and 5 °C

Ethylene “living” polymerization was conducted using the two binuclear catalysts, **3a** and **3b**, respectively. For comparison purpose, polymerization with the mononuclear catalyst **2** was also carried out as a control run. A polymerization condition with an ethylene pressure of 400 psi and a temperature of 5 °C, typical for ethylene “living” polymerization with Pd–diimine catalysts [15,16,17,18,19,20,21,22,23,24,25,26,27,28,29,30], was used. A catalyst concentration with [Pd] = 3.3 × 10^−4^ M was used for all the polymerization runs. During the polymerizations, aliquots of the polymerization solution in chlorobenzene were removed every 1 h for 6 h and quenched with Et_3_SiH prior to polymer isolation. The polymers obtained were analyzed using GPC to determine the average molecular weight and molecular weight distribution and ^1^H NMR to elucidate chain microstructure.

**Figure 3:**
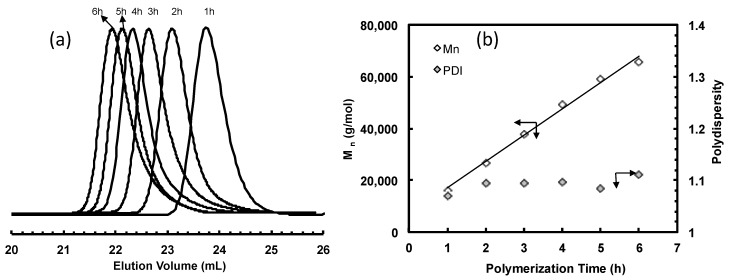
(**a**) GPC elution curves for polyethylene samples taken at different polymerization time in ethylene polymerization using **2** at 5 °C and 400 psi in chlorobenzene; and (**b**) plot of *M_n_* and *PDI* as functions of polymerization time.

Table 1 summarizes the results for ethylene polymerization using catalysts **2**. Figure 3a shows the GPC elution traces of polymer samples taken at different polymerization time during this polymerization and Figure 3b plots the *M_n_* and *PDI* vs. time. Characteristics of “livingness” of this polymerization system with **2** can be corroborated by the linear increase of *M_n_* with polymerization time. Monomodal molecular weight distribution with polydispersity below 1.11 is observed with all the samples. However, with the increase of polymerization time, *PDI* increases slightly, and a low molecular weight tail in the GPC elution trace appears and becomes more obvious, indicating slight catalyst deactivation during polymerization. The *M_n_* values shown in Table 2, which are based on polystyrene standards, are very close to those reported in the literature [15] with ethylene polymerization using **2** under the identical condition. From ^1^H NMR analyses, the polymers are all highly branched with ca. 100 branches/1000 carbons, which was resulted from the characteristic chain walking mechanism of Pd–diimine catalysts [13,31,32,33,34,37,38,39,40,41,42,43,44,45,46]. The end-capping ester group –C(O)OCH_3_ were evidenced in the ^1^H NMR spectra of all the samples. Based on the fact that each polymer chain contains one end-capping ester group, the turnover frequency (TOF) was calculated to be 309–367/h from the ^1^H NMR results of the three samples obtained within the first three hours (2-E400-1, 2-E400-2, and 2-E400-3). This TOF result is much higher compared to the literature reported data [15,16] for the same polymerization system, 216/h, which was calculated based on the weight of polymers produced per mole of catalysts employed. This difference indicates incomplete initiation or decomposition of some catalyst centers during the polymerization.

With the binding of two acrylate chelates on one molecule through the ester linkage, the binuclear catalysts **3a** and **3b** are supposed to initiate the bifunctional ethylene “living” polymerization with the simultaneous chain growth in two directions (Equation (4)). Such bifunctional “living” polymerization should theoretically yield polymers having a molecular weight twice the value of corresponding polymers obtained with the mononuclear catalyst **2** after the same polymerization time.


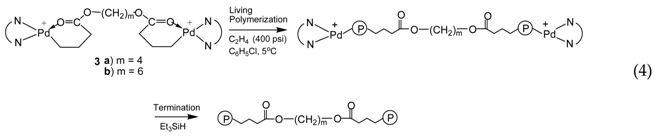


**Figure 4:**
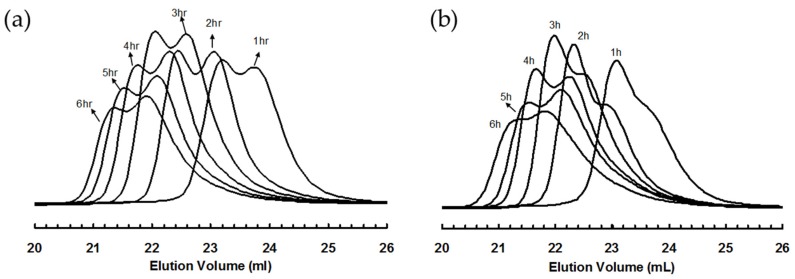
GPC elution curves for polyethylene samples taken at different polymerization time in ethylene “living” polymerization using (**a**) **3a**; and (**b**) **3b**, at 5 °C and 400 psi in cholorobenzene.

Figure 4a,b shows the GPC elution curves for the polymers obtained with catalysts **3a** and **3b**, respectively, at different polymerization times at 400 psi and 5 °C. Surprisingly, bimodal molecular weight distribution is observed for all the polymer samples obtained in polymerizations with both catalysts. Moreover, with the increase of polymerization time, the elution curves shift towards left with reduced elution volume, indicating the increase of average molecular weight with polymerization time. However, the polymers obtained have a *PDI* below 1.41 regardless of their bimodal nature. From their ^1^H NMR spectra, the polymer samples possess branching density of ca. 100 branches/1000 C, which is almost identical to the polymers synthesized with **2** at the same condition. Table 2 and Table 3 summarize *M_n_* and *PDI* values together with branching density data for these two batches of polymers synthesized with **3a** and **3b**, respectively.

**Figure 5:**
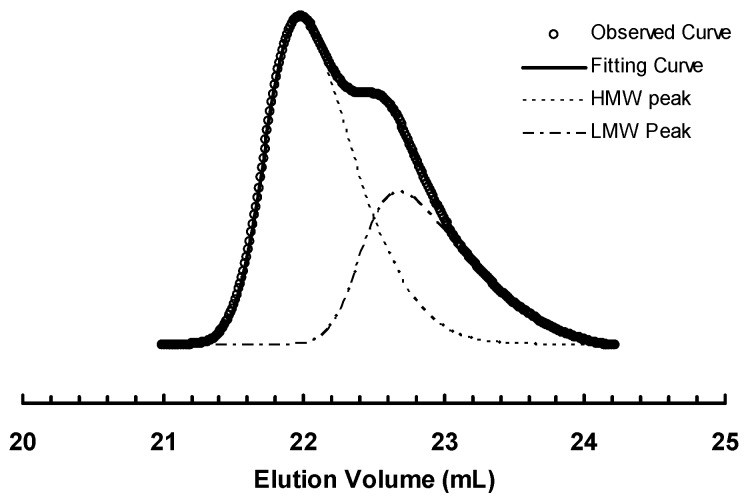
Deconvolution of GPC elution curve for sample 3b-E400-3.

The bimodal molecular weight distribution behavior indicates the presence of two different types of catalytic growing species in the polymerization systems. Deconvolutions of GPC elution curves were conducted using a PeakFit software (v. 4.12; Systat Software, San Jose, CA, USA) to retrieve the information and relationship between the molecular weight values of polymers generated by these two types of species. The empirical half-Gaussian modified Gaussian (GMG) model was applied for all the deconvolutions. All the bimodal GPC elution traces were deconvoluted into two peaks, one high molecular weight (HMW) peak and the other low molecular weight (LMW) peak. To illustrate the effectiveness of the deconvolution procedure, Figure 5 shows the two deconvoluted peaks for polymer sample 3b-E400-3, produced with **3b** after 3 h of polymerization time, and compares the resulting fitting curve with the originally observed GPC elution curve. The chromatogram is well fitted within the whole peak range from the figure.

**Figure 6:**
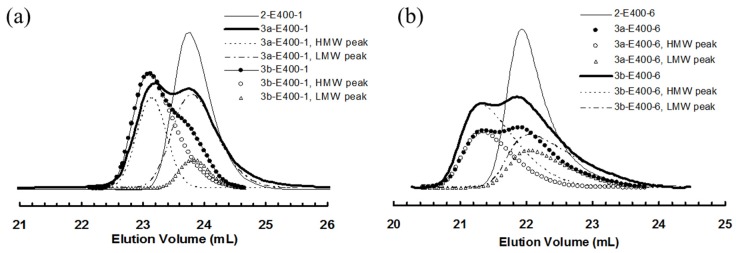
(**a**) Comparison of GPC elution curves for 2-E400-1, 3a-E400-1 and 3b-E400-1; and (**b**) comparison of GPC elution curves for 2-E400-6, 3a-E400-6 and 3b-E400-6.

Deconvolution of GPC elution curve was performed on all the bimodal polymers obtained in polymerizations with **3a** and **3b**. One unique similarity is observed with the two batches of polymers obtained with the two catalysts. Comparing any pair of polymer samples obtained with **3a** and **3b**, respectively, at the same polymerization time, it is found that their LMW peaks exhibit almost identical peak positions and so do their HMW peaks. Moreover, the peak positions for the LMW peaks are also very close to that of corresponding polymer obtained with catalyst **2** after the same polymerization time. Figure 6a,b demonstrates this similarity by comparing the elution curves and deconvoluted peaks for the three polymers obtained using the three catalysts with a polymerization time of 1 h and 6 h, respectively.

**Figure 7:**
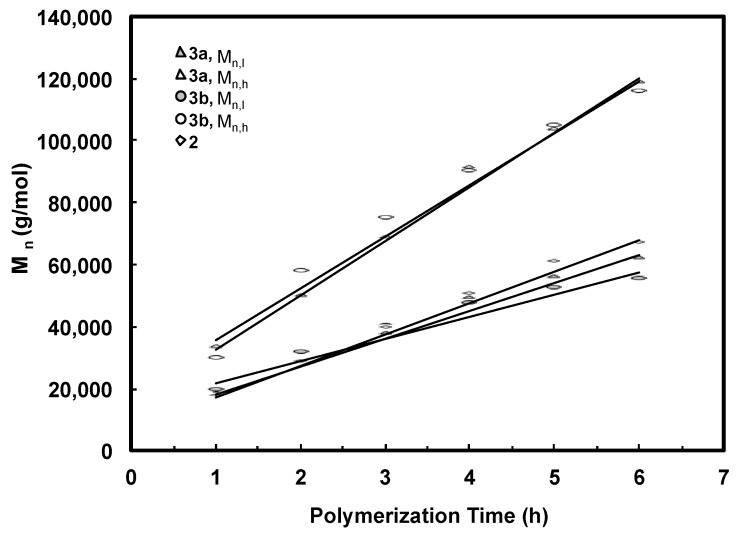
Number-average molecular weight of deconvoluted peaks as a function of polymerization time for polymers produced with catalyst **3a** and **3b** under 5 °C and 400 psi. Comparison is made with polymers prepared using catalyst **2**.

The *M_n_* and *PDI* values together with the area percentages for the fitted LMW and HMW peaks of all the bimodal polymers were calculated. These data are listed in Table 2 and Table 3. For both batches of polymers obtained with **3a** and **3b**, it is found from Table 2 and Table 3 that the *M_n_* values for both LMW and HMW peaks (*M_n,l_* and *M_n,h_*, respectively) increase with polymerization time. Figure 7 plots *M_n,l_* and *M_n,h_* as functions of the polymerization time for the two batches of polymers along with the data for polymers synthesized with catalyst **2** for comparison. Strikingly, linear dependencies of both *M_n,l_* and *M_n,h_* with time are evidenced. Moreover, the *M_n,l_* and *M_n,h_* values are almost identical for the polymers obtained with **3a** and **3b** after the same polymerization time. The *M_n,l_* values are also very close to those of polymers synthesized with catalyst **2** with the same polymerization time. More importantly, it is also found that the ratio of *M_n,h_*/*M_n,l_* for all the polymer samples obtained with **3a** and **3b** is always kept at around 2.0 (Table 2 and Table 3). Based on these evidences, we conclude that the LMW peaks correspond to polymers generated through monofunctional chain growth by monofunctional chain growing species resembling catalyst **2** and the HMW peaks represent polymers with twice molecular weight through bifunctional chain growth by the bifunctional species. Both these types of species exhibit “living” characteristics, as can be evidenced from the linear increases of both *M_n,l_* and *M_n,h_* with time. For all the polymers, the *PDI* values of their HMW and LMW peaks are low and are generally below 1.20 (Table 2 and Table 3). However, the *PDI* values of both peaks in each set of polymers increase with polymerization time. Low molecular weight tails are observed in their GPC elution traces and they increase proportionally with the polymerization time (Figure 4), indicating the occurrence of some catalyst deactivation during polymerization.

Given the high purity of **3a** and **3b** with no residual mononuclear precursor **1** or singly-chelated complexes with one unreacted pendant acrylate group observed in our characterizations above, the presence of the monofunctional species in the polymerization systems with binuclear catalysts **3a** and **3b** indicates the possible incomplete initiation/activation of some metal centers of the binuclear chelate complexes and/or deactivation/chain transfer reactions of one active site in the bifunctional species during the polymerization. Similar LMW polymer fractions resulting from mononuclear catalytic species were also observed in ethylene “living” polymerization catalyzed with trinuclear and multinuclear Pd–diimine chelate complexes reported in the earlier studies by our group [21,22].

Owing to having the identical metal center structure, all the active growing sites, regardless of the catalysts, should possess the same TOF. The monofunctional species containing only one active site should resemble the polymerization behavior of the mononuclear catalyst **2**, thus producing polymers with similar molecular weight. This also leads to the similarity observed above with *M_n,l_* and *M_n,h_* being close in the two groups of polymers obtained with **3a** and **3b**. The concentration ratio between these two types of species can be approximately reflected by the area percentages of the fitted LMW and HMW peaks, which are listed in Table 2 and Table 3 as well. For polymerization with catalyst **3a**, the area percentage of the HMW peak first increases from 39% at 1 h to 61% at 2 h, and then slowly decreases. This trend of change reflects the initial increase (showing continuous chain initiation) and the late decrease (showing catalyst deactivation) in the concentration of bifunctional growing species during the course of polymerization. However, for polymerization with **3b**, a continuous decrease in the area percentage of the LMW peak is found, indicating the chain initiation is faster with **3b**. Based on this phenomenon, we hypothesize that chain initiation might be related to the linkage length between the two Pd chelates bound together in the same binuclear complex. The longer linkage length in **3b** (two methylenes longer than **3a**) might help reduce interactions between the two metal centers and improve chain initiation. The presence of both monofunctional and bifunctional chain growing species was also observed in bifunctional propylene “living” polymerization using V(acac)_3_/AlEt_3_Cl/diene catalyst system reported by Murata et al [10].

**Figure 8:**
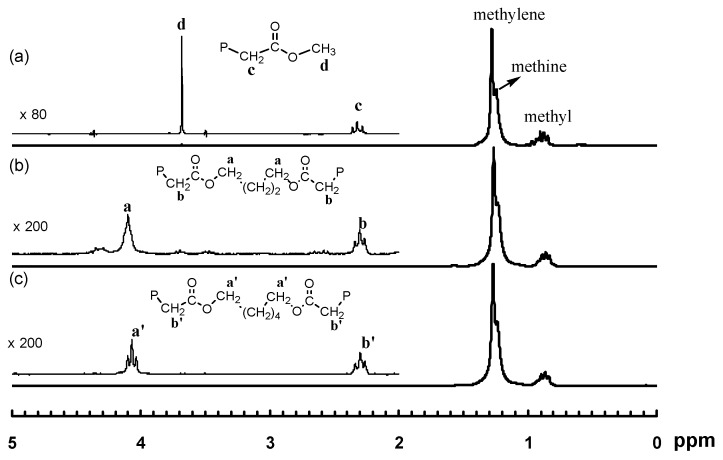
Polymer ^1^H NMR spectra for: (**a**) 2-E400-2; (**b**) 3a-E400-2; and (**c**) 3b-E400-2. All three polymers were produced after 2 h of polymerization time.

**Scheme 3:**
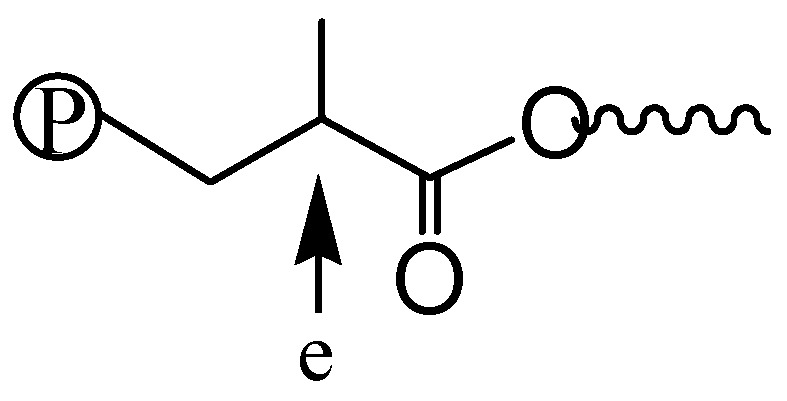
Chain microstructure resulting from the five-membered chelate isomer.

Figure 8 shows the ^1^H NMR spectra of three polymers, 2-E400-2, 3a-E400-2 and 3b-E400-2, synthesized with **2**, **3a** and **3b**, respectively, with 2 h of polymerization time. The end-capping methyl ester functionality is evidenced in 2-E400-2 (Figure 8a). In both 3a-E400-2 and 3b-E400-2, an ester linkage is found with two triplet methylene resonances (a and b for 3a-E400-2 in Figure 8b); a’ and b’ for 3b-E400-2 in Figure 8c centered at 4.1 and 2.3 ppm, respectively. We have shown above that the isomeric five-membered chelate structure (Scheme 2) exists in both **3a** and **3b** at a significant percentage of ~13%. If this chelate could also initiate ethylene “living” polymerization, the resulting polymer would possess the unique microstructure shown in Scheme 3, where there is a methine group next to the ester functionality. The proton of this methine group (e in Scheme 3) in this microstructure should have a multiplet centered at 2.5 ppm in the ^1^H NMR spectrum. However, this resonance was not found in the ^1^H NMR spectra (with at least 10,000 scans) of all the polymers analyzed. This microstructure was also not reported in the literature [15,16] with polymers obtained in ethylene “living” polymerization with catalyst **2**, which was reported to have ~11% of the above five-membered chelate isomer [36]. These results indicate that the five-membered chelates are possibly incapable of initiating ethylene “living” polymerization. If this is the case, it is another factor contributing to the presence of monofunctional chain growth in the polymerizations with **3a** and **3b**.

**Figure 9:**
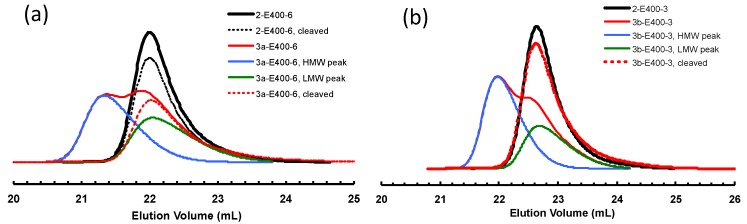
Comparison of GPC elution curves of polymer samples before and after cleavage: (**a**) samples 2-E400-6 and 3a-E400-6; and (**b**) samples 2-E400-3 and 3b-E400-3.

It is envisaged that, if the ester linkage is cleaved, the molecular weight of the polymers obtained by bifunctional chain growth with **3a** and **3b** will be halved considering the linkage being centered in the middle of the chain. Differently, for polymers obtained by monofunctional chain growth, their molecular weights should only be negligibly affected by cleavage due to the location of the ester linkage close to the chain end. It is thus expected that, after cleavage, the original bimodal polymers by **3a** and **3b** should exhibit a single GPC peak, which should be almost identical to the original LMW peak and to the GPC peak of corresponding polymer by **2** after the same polymerization time. This hypothesis is verified by conducting cleavage experiments on some selected polymer samples by hydrolysis of the ester linkages using KOH in a mixture of THF and methanol [53]. The resulting polymers after hydrolysis were characterized by GPC. Table 4 lists these samples and their molecular weights before and after the cleavage. Figure 9a shows the effects of hydrolysis on the GPC elution curves of two polymers, 2-E400-6 (as a control sample) and 3a-E400-6, synthesized with **2** and **3a**, respectively, with 6 h of polymerization time. For polymer 2-E400-6 grown from the monofunctional catalyst **2**, no obvious change in the position and width of GPC elution curves can be found before and after the cleavage, and the changes in *M_n_* and *PDI* are very small (Table 4). This confirms that the polymer having a hydrocarbon backbone is stable with negligible degradation in the basic hydrolysis condition. Differently, for polymer 3a-E400-6, the cleaved sample exhibits a monomodal GPC curve in sharp contrast to the original bimodal curve. A significant 30% drop in *M_n_* is found after the cleavage. Moreover, the *M_n_* and *PDI* values for the cleaved sample are very similar to those of the LMW peak and polymer 2-E400-6. This evidence further proves our conclusion above that the HMW peaks correspond to polymers obtained by bifunctional chain growth while the LMW peaks represent polymers from monofunctional chain growth. Figure 9b shows the effect of cleavage on polymer 3b-E400-3. Similarly, the cleavage resulted in a monomodal GPC curve with *M_n_* and *PDI* similar to those of corresponding LMW peak and polymer 2-E400-3.

### 3.3. Ethylene “Living” Polymerization with **3a** at 100 psi and 5 °C

For ethylene polymerization with catalyst **2** at 5 °C, it has been reported by Brookhart et al. that the “living” polymerization characteristics is maintained within an ethylene pressure range of 100–400 psi and a loss in the livingness occurs at a lowered ethylene pressure of 1 atm due to the reduced chain initiation rate relative to chain propagation [15]. Moreover, it was also demonstrated that TOF of polymerization and *M_n_* values of the resulting polymers were independent of ethylene pressure within the pressure range of 100–400 psi. These are due to the facts that the alkyl olefin complex, (diimine)Pd(C_2_H_4_)R^+^, is the catalyst resting state and the migratory insertion is the rate-controlling step [15]. Ethylene polymerization with catalyst **3a** was also conducted under a pressure of 100 psi and 5 °C in this work to examine the pressure effects on the polymerization behavior using this binuclear catalyst. Table 5 summarizes the polymerization results.

**Figure 10:**
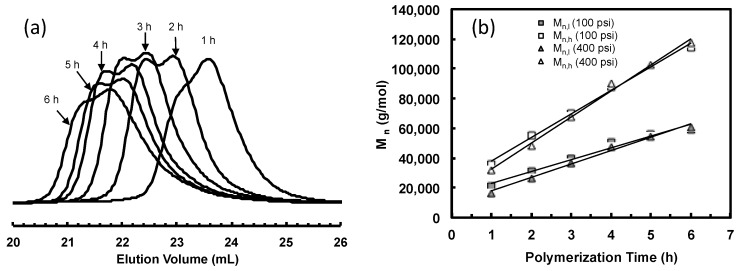
(**a**) GPC elution curves for polyethylene samples taken at different polymerization time in ethylene polymerization using **3a** at 5 °C and 100 psi in chlorobenzene; and (**b**) *M_n,l_* and *M_n,h_* values of the deconvoluted peaks as a function of polymerization time for polymers produced at 100 psi and 400 psi using **3a**.

Bimodal GPC curves are also featured with the polymers prepared under this pressure, indicating again the presence of both monofunctional and bifunctional chain growing species. Figure 10a shows the evolution of GPC elution curves with the increase of polymerization time. An increase of overall *M_n_* with polymerization time is evident (Table 5). The bimodal GPC curves were also deconvoluted into the LMW and HMW peaks. Table 5 lists the *M_n,l_*, *M_n,h_*, and *PDI* values of the deconvoluted peaks together with their relative area percentages. Figure 10b plots both *M_n,l_* and *M_n,h_*, vs. polymerization time along with the data for polymers obtained above with **3a** at 400 psi. Within the experimental error of GPC measurements, the *M_n,l_* and *M_n,h_* values are very close between polymers obtained with the same polymerization time in these two runs at different pressures. Consistent to the literature results with catalyst **2**, this also proves that ethylene pressure does not affect TOF and *M_n_*. Moreover, the linear dependences of both *M_n,l_* and *M_n,h_* on polymerization time indicates the “living” characteristics of both types of initiating species. The ratio of *M_n,h_*/*M_n,l_* is maintained at ~2. Similar to the trend observed in the run at 400 psi, the relative area percentage for the HMW peak initially increases from 22% at 1 h to 59% at 3 h and then decreases in this run, indicating the initial increase and subsequent decrease in the relative concentration of the bifunctional chain growing species. However, comparing relative area percentages of the HMW peaks at 1 h, the value is significantly higher (39%) in the run at 400 psi. This suggests the slower chain initiation at the reduced ethylene pressure of 100 psi in the very beginning of polymerization.

### 3.4. “Living” Polymerization of 1-Hexene at 0 °C

**Figure 11:**
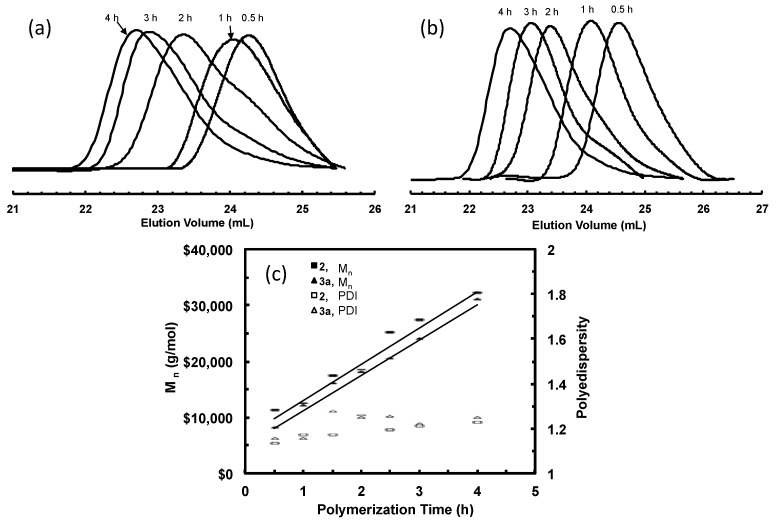
GPC elution curves of poly(1-hexene)s produced at different polymerization time using: (**a**) **2**; and (**b**) **3a**, at 0 °C and [1-hexene] = 1.15 M. (**c**) Plot of *M_n_* and *PDI* for poly(1-hexene)s produced with **2** and **3a** as functions of polymerization time.

1-Hexene “living” polymerizations were also carried out with both catalysts **3a** and **2** (as a control run) at a 1-hexene concentration of 1.15 M and at 0 °C to investigate the efficiency of the binuclear catalyst for “living” polymerization of α-olefins. Table 6 summarizes the *M_n_* and *PDI* data for polymers obtained at different polymerization time in both runs. The “living” behavior was achieved with catalyst **2**. Figure 11a shows the evolution of polymer GPC elution curves with the polymerization time and Figure 11c plots *M_n_* and *PDI* vs. polymerization time. A good linear increase of *M_n_* with time can be evidenced and *PDI* values remain below 1.23. Figure 11b shows the evolution of GPC curves for polymers obtained with **3a**. Surprisingly, unlike the bimodal polyethylenes obtained above, all the poly(1-hexene) samples show monomodal GPC curves, which suggests the presence of a single chain growing species. It is also found that the poly(1-hexene) samples obtained in both runs with **2** and **3a** have very close *M_n_* values. This leads us to believe that the chain growing species in 1-hexene polymerization with **3a** should be the monofunctional species and the bifunctional species should be absent. Figure 11c plots *M_n_* and *PDI* values for the polymers by **3a**. A linear increase of *M_n_* with time is clear and *PDI* remains below 1.26, showing the “living” characteristics in the polymerization with **3a**. However, for polymers produced with both **2** and **3a**, slight increases of *PDI* values with time are observed, indicating the presence of catalyst deactivation and/or chain transfer reactions.

**Figure 12:**
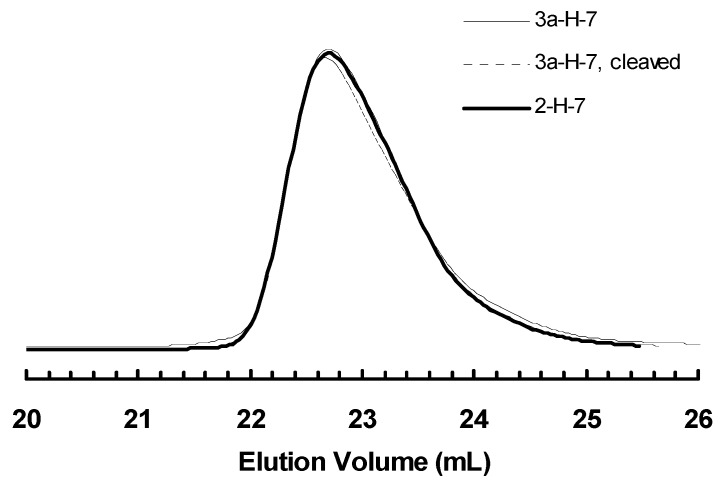
GPC elution curve of cleaved sample 3a-H-7, in comparison with those of 3a-H-7 and 2-H-7.

Polymer cleavage by hydrolysis under basic condition was also carried out on one poly(1-hexene) sample, 3a-H-7, obtained with **3a** at 3 h. Figure 12 shows the GPC curves of this polymer before and after cleavage together with that for polymer obtained with **2** at 3 h, 2-H-7, for comparison. One can see that the change in the curves is negligible for 3a-H-7 before and after the cleavage and the two polymers, 3a-H-7 and 2-H-7, exhibit almost identical elution curves. This cleavage result further supports that 1-hexene polymerization with **3a** was carried out by the monofunctional species alone. However, the precise reason to the absence of the bifunctional initiating species for this binuclear catalyst in 1-hexene polymerization is not known. We hypothesize that, in 1-hexene polymerization, possibly the initiation of one chelate complex disables the initiation of the other one bound on the same molecule. Further research needs to be conducted to elucidate the exact mechanism.

## 4. Conclusions

Two novel binuclear Pd–diimine acrylate chelate complexes, **3a** and **3b**, were synthesized in this work by reacting the acetonitrile adduct **1** with two diacrylates, 1,4-butanediol diacrylate and 1,6-hexanediol diacrylate, respectively. The binuclear structure of the catalysts was confirmed using NMR, mass spectrometry, and single crystal XRD measurements. Both binuclear catalysts initiated successfully the “living” polymerization of ethylene at 5 °C and under ethylene pressure of 400 psi. However, polymers with bimodal molecular weight distribution were obtained with both binuclear catalysts in contrast to the monomodal polymers obtained with the mononuclear catalyst **2**. Deconvolution of the bimodal GPC curves and polymer cleavage experiments prove the existence of both bifunctional and monofunctional chain growing species with the bifunctional species producing polymers with a molecular weight twice that of polymers by monofunctional species. Both species exhibit “living” characteristics, leading to linear increase of polymer molecular weight with time. Characteristics of “living” polymerization were also observed with **3a** at 5 °C and under a reduced pressure of 100 psi. The polymers obtained also possess bimodal molecular weight distribution. However, the decrease of pressure from 400 psi to 100 psi does not change the *M_n_* values of polymers obtained with both types of chain growing species. Catalyst **3a** also initiated successfully the “living” polymerization of 1-hexene with linear increase of polymer molecular weight with time. However, the polymers produced exhibit monomodal molecular weight distribution, showing the sole presence of monofunctional chain growing species.

## Figures and Tables

**Table 1:** Ethylene polymerization using **2** at 5 °C and 400 psi in chlorobenzene ^a^.

Sample	Time (h)	*M_n_* ^b^ (kg/mol)	*PDI* ^b^	Branches ^c^ (/1000 C)
2-E400-1	1	16	1.06	101
2-E400-2	2	27	1.09	100
2-E400-3	3	38	1.09	105
2-E400-4	4	49	1.10	98
2-E400-5	5	59	1.08	101
2-E400-6	6	65	1.11	103

^a^ Catalyst **2** amount: 0.085 g (0.1 mmol); Total volume: 300 mL; ^b^ Molecular weight data were determined by GPC vs. polystyrene standards; ^c^ Total branching density was determined by ^1^H NMR in CDCl_3_ at ambient temperature.

**Table 2:** Ethylene polymerization using **3a** at 5 °C and 400 psi in chlorobenzene ^a^.

Sample	Time (h)	*M_n_* ^b^ (kg/mol)	*PDI* ^b^	Branches ^c^/1000 C	High Molecular Weight Peak ^d^		Low Molecular Weight Peak ^d^		*M_n,h_*/*M_n,l_*	Peak Area% (*h*/*l*)
					*M_n,h_* (kg/mol)	*PDI_h_*	*M_n,l_* (kg/mol)	*PDI_l_*		
3a-E400-1	1	21	1.21	99	32	1.04	17	1.10	1.9	39/61
3a-E400-2	2	33	1.21	98	48	1.09	27	1.09	1.8	61/39
3a-E400-3	3	45	1.24	100	67	1.09	36	1.12	1.9	60/40
3a-E400-4	4	56	1.25	100	90	1.08	48	1.12	1.9	52/48
3a-E400-5	5	66	1.26	106	102	1.10	55	1.13	1.9	44/56
3a-E400-6	6	77	1.25	101	118	1.13	61	1.15	1.9	43/57

^a^ Catalyst **3a** amount: 0.085 g (0.05 mmol); total volume: 300 mL; ^b^ Molecular weight data were determined by GPC vs. polystyrene standards; ^c^ Total branching density was determined by using ^1^H NMR in CDCl_3_ at ambient temperature; ^d^ Deconvoluted GPC peaks by using a PeakFit software (v. 4.12) with the GMG model.

**Table 3:** Ethylene polymerization using **3b** at 5 °C and 400 psi in chlorobenzene ^a^.

Sample	Time (h)	*M_n_* ^b^ (kg/mol)	*PDI* ^b^	Branches ^c^/1000 C	High Molecular Weight Peak ^d^		Low Molecular Weight Peak ^d^		*M_n,h_*/*M_n,l_*	Peak Area% (*h*/*l*)
					*M_n,h_* (kg/mol)	*PDI_h_*	*M_n,l_* (kg/mol)	*PDI_l_*		
3b-E400-1	1	22	1.20	97	29	1.09	18	1.04	1.6	85/15
3b-E400-2	2	39	1.22	98	56	1.06	30	1.07	1.9	67/33
3b-E400-3	3	51	1.19	98	73	1.08	38	1.11	1.9	65/35
3b-E400-4	4	55	1.31	100	89	1.10	46	1.13	1.9	62/38
3b-E400-5	5	60	1.32	99	103	1.12	51	1.18	2.0	43/57
3b-E400-6	6	68	1.41	101	114	1.14	54	1.22	2.1	44/56

^a^ Catalyst **3b** amount: 0.085 g (0.05 mmol); total volume: 300 mL; ^b^ Molecular weight data were determined by GPC vs. polystyrene standards; ^c^ Total branching density was determined by using ^1^H NMR in CDCl_3_ at ambient temperature; ^d^ Deconvoluted GPC peaks by using a PeakFit software (v. 4.12) with the GMG model.

**Table 4:** Effect of cleavage on polymer molecular weight and distribution ^a^.

Sample	Before Cleavage		After Cleavage		Percentage Drop in *M_n_*
	*M_n_* (kg/mol)	*PDI*	*M_n_* (kg/mol)	*PDI*	
2-E400-6	65	1.11	61	1.13	6
3a-E400-6	77	1.25	54	1.17	30
3b-E400-3	51	1.19	36	1.10	29
3b-E400-5	71	1.32	53	1.14	25

^a^ Polymer molecular weight data were determined by using GPC vs. polystyrene standards.

**Table 5:** Ethylene polymerization using **3a** at 5 °C and 100 psi in chlorobenzene ^a^.

Sample	Time (h)	*M_n_* ^b^ (kg/mol)	*PDI* ^b^	High Molecular Weight Peak ^d^		Low Molecular Weight Peak ^d^		*M_n,h_*/*M_n,l_*	Peak Area% (h/l)
				*M_n,h_* (kg/mol)	*PDI_h_*	*M_n,l_* (kg/mol)	*PDI_l_*		
3a-E100-1	1	21	1.22	36	1.03	21	1.09	1.7	22/78
3a-E100-2	2	35	1.21	55	1.05	31	1.07	1.8	49/51
3a-E100-3	3	47	1.27	70	1.08	40	1.09	1.8	59/41
3a-E100-4	4	57	1.31	88	1.09	50	1.09	1.8	60/40
3a-E100-5	5	60	1.32	103	1.11	56	1.12	1.8	53/47
3a-E100-6	6	69	1.41	114	1.13	59	1.19	1.9	24/76

^a^ Catalyst **3a** amount: 0.085 g (0.05 mmol); total volume: 300 mL; ^b^ Molecular weight data were determined by GPC vs. polystyrene standards; ^c^ Total branching density was determined by using ^1^H NMR in CDCl_3_ at ambient temperature; ^d^ Deconvoluted GPC peaks by using a PeakFit software (v. 4.12) with the GMG model.

**Table 6:** 1-Hexene polymerization using **2** and **3a** in CH_2_Cl_2_^a^.

Sample	Catalyst	Time (h)	*M_n_* ^b^ (kg/mol)	*PDI* ^b^
2-H-1	2	0.5	11	1.11
2-H-2	2	1	12	1.15
2-H-3	2	1.5	17	1.15
2-H-4	2	2	18	1.23
2-H-5	2	2.5	24	1.17
2-H-6	2	3	27	1.19
2-H-7	2	4	32	1.21
3a-H-1	3a	0.5	8	1.11
3a-H-2	3a	1	12	1.10
3a-H-3	3a	1.5	15	1.16
3a-H-4	3a	2	17	1.23
3a-H-5	3a	2.5	20	1.24
3a-H-6	3a	3	23	1.20
3a-H-7	3a	3	31	1.23

^a^ Catalyst amount: **2**, 0.085 g (0.1 mmol); **3a**, 0.086 g (0.05 mmol). 1-Hexene amount: 15 mL (1.15 M in concentration). Total volume: 105 mL; Temperature: 0 °C; ^b^ Molecular weight data were determined by GPC vs. polystyrene standards.

## Supplementary Materials

Supplementary materials are available online at www.mdpi.com/2073-4360/9/7/282/s1.
