# Supplementary material for: “Living” Polymerization of Ethylene and 1-Hexene Using Novel Binuclear Pd–Diimine Catalysts
Source: Polymers (Basel). 2017 Jul 15;9(7):282. doi: 10.3390/polym9070282 (PMC6432338; doi:10.3390/polym9070282)
Supplement: Supplementary file 1 [file polymers-09-00282-s001.pdf]

Supplementary Materials: "Living" Polymerization of Ethylene and 1-Hexene Using Novel Binuclear Pd–Diimine Catalysts

Jianding Ye and Zhibin Ye

Contents:

Figure S1 ^{13}C NMR spectrum for **3a**.

Figure S2 ^{13}C NMR spectrum for **3b**.

Figure S3 ESI MS spectra (positive ion mode) of **3b**.

Figure S4 Thermal ellipsoid of **3b**.

Figure S5 Stick-ball diagram of **3b**.

Table S1 Crystal data and structure refinement for **3b**.

Table S2 Atomic coordinates and equivalent isotropic displacement parameters for **3b**

Table S3 Bond lengths and angles for **3b**.

Table S4 Anisotropic displacement parameters for **3b**.

Table S5 Hydrogen coordinates and isotropic displacement parameters for **3b**.

Table S6 Torsion angles for **3b**.

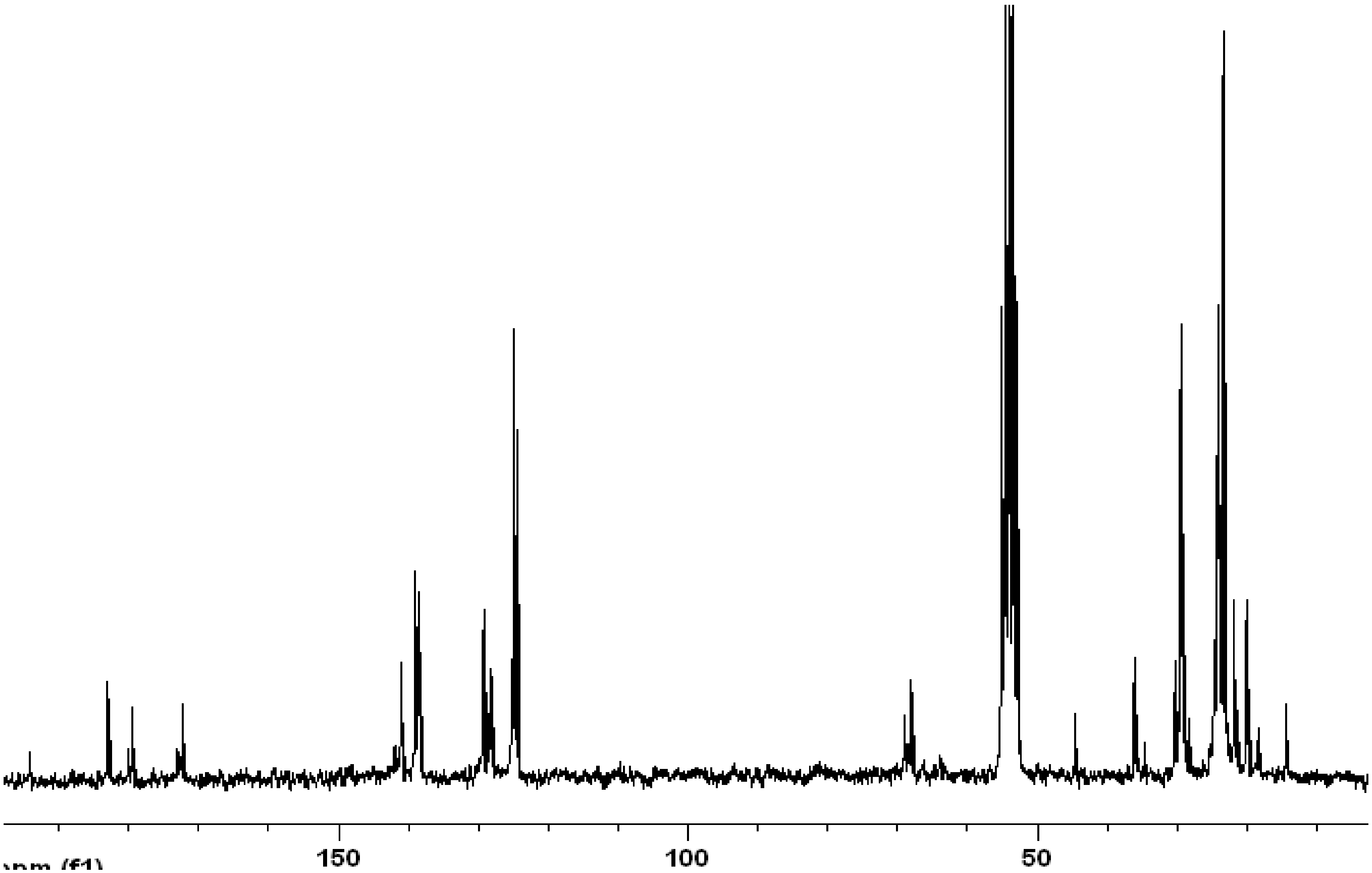

Figure S1 ^{13}C NMR spectrum for **3a** in CD_2Cl_2.

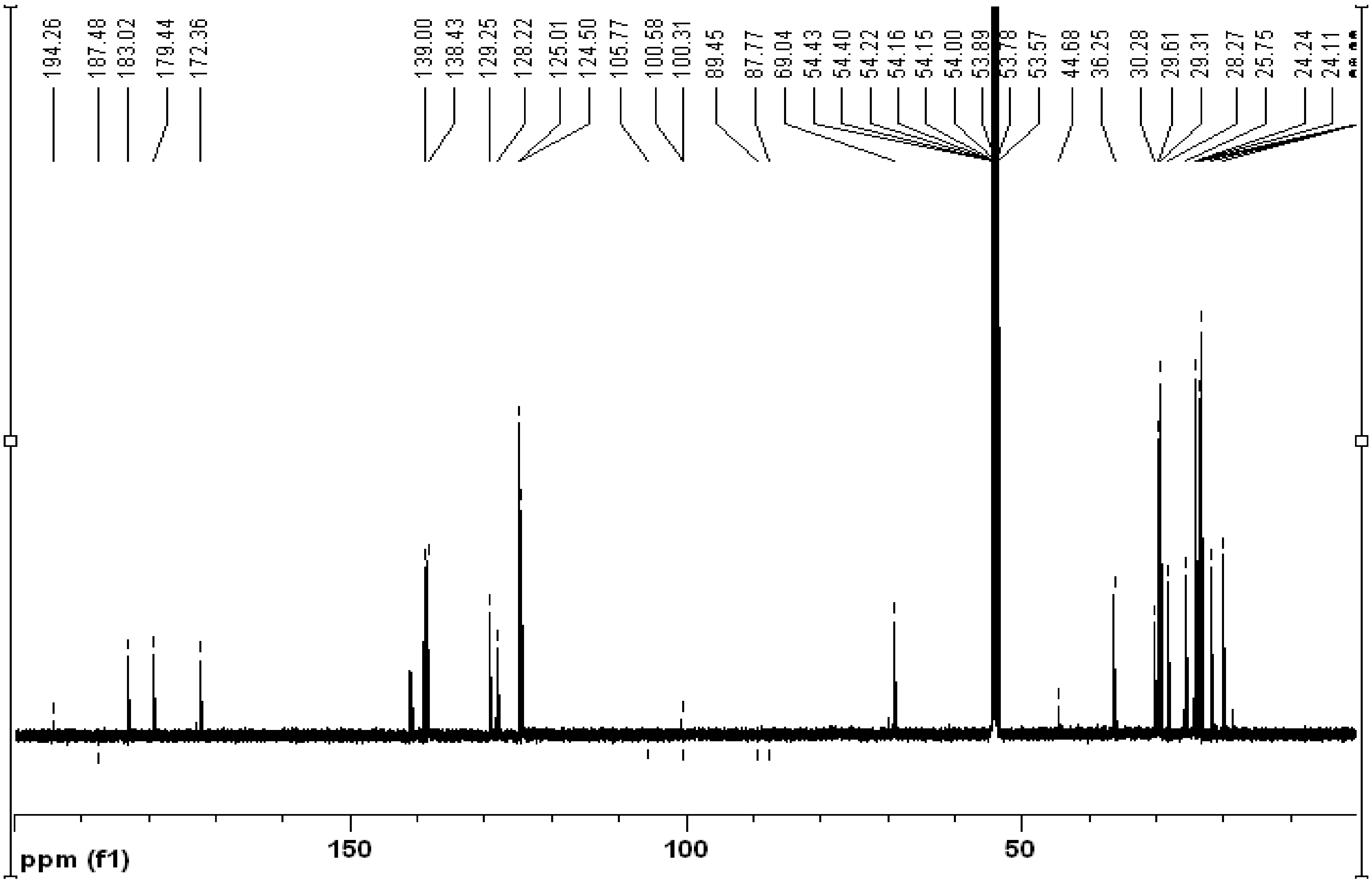

Figure S2 ^{13}C NMR spectrum for **3b** in CD_2Cl_2.

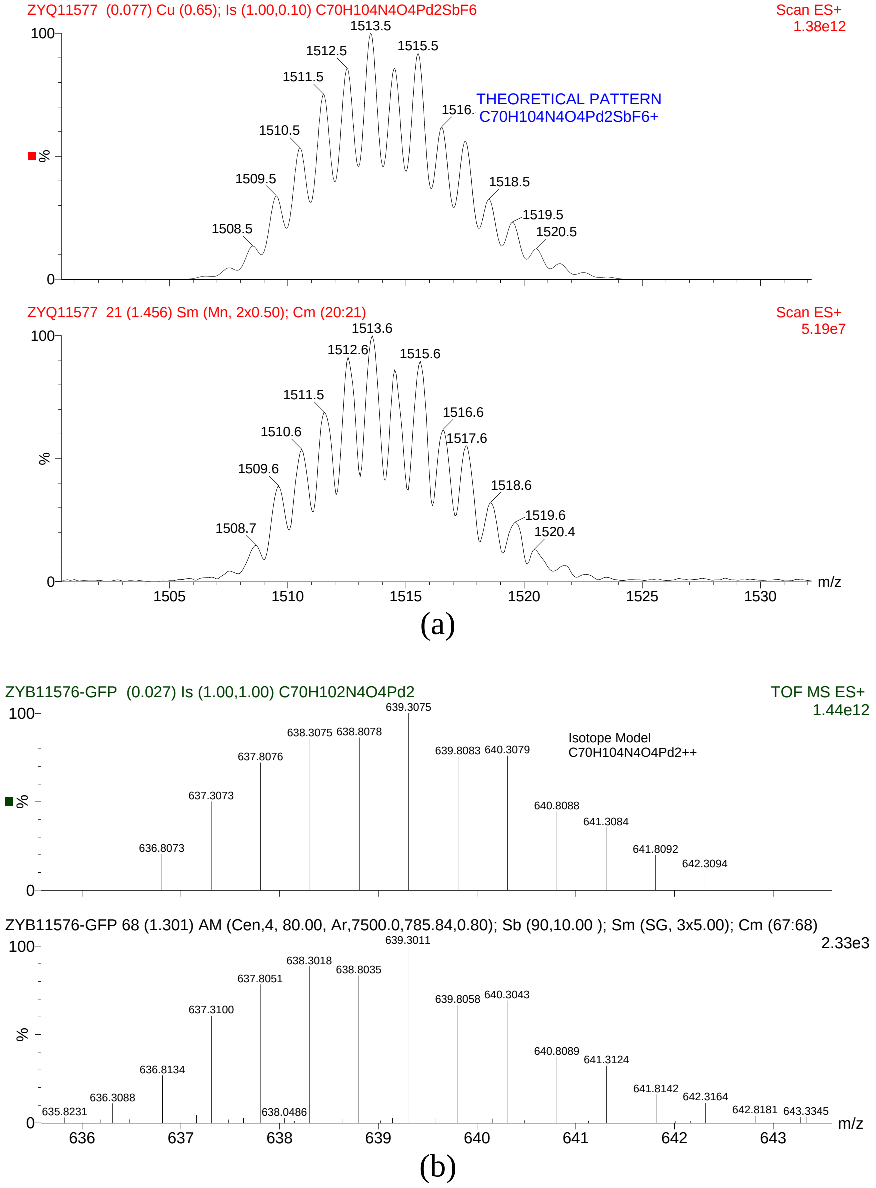

Figure S3 ESI MS spectra (positive ion mode) of **3b**: (a) narrow band scan of singly-charged region; (b) narrow band scan of doubly-charged region.

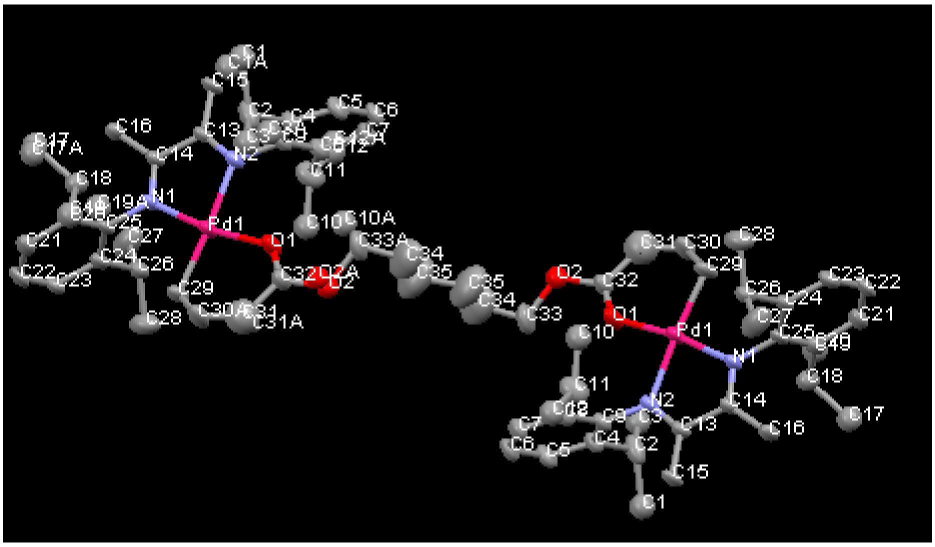

Figure S4 Thermal ellipsoid (30% probability) of **3b**. Hydrogen atoms, solvent molecule, SbF_6^- counterions, and the disordered parts of the structure are omitted.

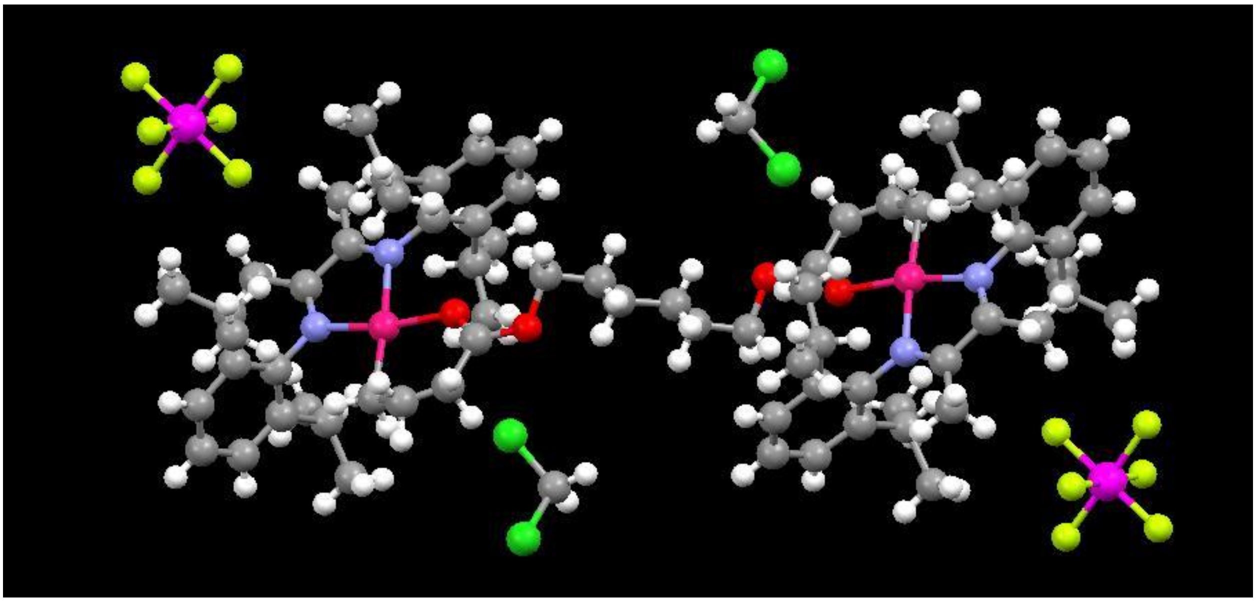

Figure S5 Stick-ball diagram for **3b**.

Table S1 Crystal data and structure refinement for **3b**.

Identification code	**3b**	
Empirical formula	$C_{72} H_{108} Cl_4 F_{12} N_4 O_4 Pd_2 Sb_2$	
Formula weight	1919.72	
Temperature	173(2) K	
Wavelength	0.71073 Å	
Crystal system	Monoclinic	
Space group	$P2_1/n$	
Unit cell dimensions	a = 10.7600(7) Å	α= 90°.
	b = 21.5931(14) Å	β= 100.2870(10)°.
	c = 18.4548(12) Å	γ = 90°.
Volume	4218.9(5) $Å^3$	
Z	2	
Density (calculated)	1.511 Mg/m^3	
Absorption coefficient	1.251 mm^{-1}	
F(000)	1940	
Crystal size	0.42 x 0.40 x 0.04 mm^3	
Theta range for data collection	1.89 to 32.61°.	
Index ranges	-16<=h<=5, -32<=k<=32, -27<=l<=27	
Reflections collected	109326	
Independent reflections	15333 [R(int) = 0.0565]	
Completeness to theta = 32.61°	99.5 %	
Max. and min. transmission	0.9517 and 0.6216	
Refinement method	Full-matrix least-squares on F^2	
Data / restraints / parameters	15333 / 46 / 495	
Goodness-of-fit on F^2	0.894	
Final R indices [I>2sigma(I)]	R1 = 0.0608, wR2 = 0.1823	
R indices (all data)	R1 = 0.1104, wR2 = 0.2326	
Largest diff. peak and hole	1.892 and -0.963 e.$Å^{-3}$	

Table S2 Atomic coordinates (x 10^4) and equivalent isotropic displacement parameters ($Å^2$x 10^3) for **3b**. U(eq) is defined as one third of the trace of the orthogonalized U^{ij} tensor.

	x	y	z	U(eq)
Pd(1)	2730(1)	-2337(1)	937(1)	61(1)
O(1)	3506(3)	-3198(2)	1176(3)	83(1)
O(2A)	3846(11)	-4095(5)	1728(8)	69(1)
O(2)	3677(7)	-4182(3)	1393(5)	69(1)
N(1)	2246(3)	-1450(2)	695(2)	54(1)
N(2)	4401(3)	-1972(2)	630(2)	56(1)
C(1)	7274(12)	-1641(5)	2242(6)	90(1)
C(1A)	6861(14)	-1566(4)	2215(5)	90(1)
C(2)	6134(6)	-2114(4)	2001(4)	91(2)
C(3)	5791(14)	-2455(6)	2559(4)	90(1)
C(3A)	6372(16)	-2630(6)	2637(3)	90(1)
C(4)	6345(4)	-2426(2)	1294(2)	61(1)
C(5)	7375(4)	-2815(3)	1293(3)	70(1)
C(6)	7568(5)	-3099(3)	661(3)	71(1)
C(7)	6742(5)	-3002(3)	9(3)	70(1)
C(8)	5695(4)	-2618(3)	-20(2)	62(1)
C(9)	5518(4)	-2334(2)	621(3)	52(1)
C(10)	3594(8)	-2910(7)	-771(6)	90(1)
C(10A)	4194(12)	-3238(6)	-941(6)	90(1)
C(11)	4720(7)	-2543(4)	-728(3)	93(2)
C(12)	5258(17)	-2425(12)	-1384(3)	90(1)
C(12A)	5450(20)	-2373(11)	-1369(4)	90(1)
C(13)	4328(3)	-1396(2)	456(3)	52(1)
C(14)	3107(4)	-1096(2)	509(3)	55(1)
C(15)	5335(4)	-1030(2)	198(3)	65(1)
C(16)	2944(5)	-419(2)	367(3)	64(1)
C(17)	2233(15)	-183(5)	2329(5)	90(1)
C(17A)	2106(16)	-256(5)	2464(6)	90(1)
C(18)	2025(6)	-869(3)	2078(3)	73(1)
C(19)	1602(14)	-1220(6)	2763(4)	90(1)
C(19A)	2124(13)	-1416(5)	2578(5)	90(1)
C(20)	925(4)	-909(2)	1439(3)	60(1)
C(21)	-269(5)	-682(3)	1501(4)	71(1)
C(22)	-1275(4)	-717(3)	917(4)	75(2)
C(23)	-1135(4)	-999(3)	284(4)	69(1)
C(24)	16(4)	-1251(2)	187(2)	59(1)
C(25)	1040(3)	-1187(2)	772(3)	53(1)
C(26)	152(5)	-1550(3)	-543(3)	67(1)
C(27)	280(10)	-1062(3)	-1120(3)	122(3)
C(28)	-951(8)	-1985(3)	-813(3)	107(3)
C(29)	1057(5)	-2611(2)	1190(4)	74(2)

C(30)	1174(6)	-3145(3)	1723(5)	92(2)
C(30A)	1174(6)	-3145(3)	1723(5)	92(2)
C(31A)	1710(9)	-3727(4)	1465(8)	39(2)
C(31)	1850(30)	-3602(13)	1682(16)	168(11)
C(32)	3080(5)	-3637(2)	1405(3)	67(1)
C(33)	4998(7)	-4172(4)	1381(5)	103(2)
C(33A)	4998(7)	-4172(4)	1381(5)	103(2)
C(34)	5319(17)	-4687(7)	985(7)	188(7)
C(35)	4759(19)	-4739(7)	211(8)	212(8)
Sb(1)	6029(1)	423(1)	2184(1)	64(1)
F(1)	4474(4)	777(2)	1803(3)	98(1)
F(2)	7576(4)	58(3)	2521(3)	126(2)
F(3)	5252(7)	-351(3)	2072(8)	92(3)
F(4)	5664(12)	479(6)	3121(4)	120(5)
F(5)	6748(9)	1190(4)	2287(6)	102(4)
F(6)	6354(9)	351(6)	1252(4)	95(3)
Sb(1A)	6029	423	2184	64
F(1A)	4474(4)	777(2)	1803(3)	98(1)
F(2A)	7576(4)	58(3)	2521(3)	126(2)
F(3A)	5258(12)	-273(6)	2478(13)	199(12)
F(4A)	6030(20)	790(12)	3075(7)	350(30)
F(5A)	6782(11)	1093(6)	1845(16)	290(20)
F(6A)	5954(16)	23(9)	1297(5)	138(7)
C(36)	183(13)	-4400(6)	-871(7)	167(5)
Cl(1)	-1176(5)	-4402(3)	-472(3)	159(3)
Cl(2)	1056(14)	-3744(5)	-638(5)	238(7)
C(36A)	183(13)	-4400(6)	-871(7)	167(5)
Cl(1A)	50(30)	-3612(7)	-740(12)	302(15)
Cl(2A)	1340(30)	-4710(15)	-202(15)	540(40)

Table S3 Bond lengths [Å] and angles [°] for **3b**.

Pd(1)-N(1)	2.014(3)
Pd(1)-C(29)	2.027(4)
Pd(1)-O(1)	2.052(3)
Pd(1)-N(2)	2.131(3)
O(1)-C(32)	1.165(6)
O(2A)-C(32)	1.354(12)
O(2)-C(32)	1.343(8)
O(2)-C(33)	1.426(10)
N(1)-C(14)	1.294(5)
N(1)-C(25)	1.448(5)
N(2)-C(13)	1.282(6)
N(2)-C(9)	1.438(5)
C(1)-C(2)	1.598(11)
C(1)-H(1A)	0.9800
C(1)-H(1B)	0.9800
C(1)-H(1C)	0.9800
C(1A)-C(2)	1.435(11)
C(1A)-H(1AA)	0.9800
C(1A)-H(1AB)	0.9800
C(1A)-H(1AC)	0.9800
C(2)-C(3)	1.368(11)
C(2)-C(4)	1.522(7)
C(2)-C(3A)	1.605(11)
C(2)-H(2A)	1.0000
C(3)-H(3A)	0.9800
C(3)-H(3B)	0.9800
C(3)-H(3C)	0.9800
C(3A)-H(3AA)	0.9800
C(3A)-H(3AB)	0.9800
C(3A)-H(3AC)	0.9800
C(4)-C(5)	1.392(7)
C(4)-C(9)	1.406(6)
C(5)-C(6)	1.365(8)
C(5)-H(5A)	0.9500
C(6)-C(7)	1.379(8)
C(6)-H(6A)	0.9500
C(7)-C(8)	1.392(7)
C(7)-H(7A)	0.9500
C(8)-C(9)	1.375(7)
C(8)-C(11)	1.531(7)
C(10)-C(11)	1.439(11)
C(10)-H(10A)	0.9800
C(10)-H(10B)	0.9800
C(10)-H(10C)	0.9800
C(10A)-C(11)	1.629(12)
C(10A)-H(10D)	0.9800
C(10A)-H(10E)	0.9800
C(10A)-H(10F)	0.9800

C(11)-C(12)	1.455(12)
C(11)-C(12A)	1.573(14)
C(11)-H(11A)	1.0000
C(12)-H(12A)	0.9800
C(12)-H(12B)	0.9800
C(12)-H(12C)	0.9800
C(12A)-H(12D)	0.9800
C(12A)-H(12E)	0.9800
C(12A)-H(12F)	0.9800
C(13)-C(14)	1.485(5)
C(13)-C(15)	1.486(5)
C(14)-C(16)	1.490(6)
C(15)-H(15A)	0.9800
C(15)-H(15B)	0.9800
C(15)-H(15C)	0.9800
C(16)-H(16A)	0.9800
C(16)-H(16B)	0.9800
C(16)-H(16C)	0.9800
C(17)-C(18)	1.557(11)
C(17)-H(17A)	0.9800
C(17)-H(17B)	0.9800
C(17)-H(17C)	0.9800
C(17A)-C(18)	1.497(11)
C(17A)-H(17D)	0.9800
C(17A)-H(17E)	0.9800
C(17A)-H(17F)	0.9800
C(18)-C(19A)	1.491(10)
C(18)-C(20)	1.517(7)
C(18)-C(19)	1.607(10)
C(18)-H(18A)	1.0000
C(19)-H(19A)	0.9800
C(19)-H(19B)	0.9800
C(19)-H(19C)	0.9800
C(19A)-H(19D)	0.9800
C(19A)-H(19E)	0.9800
C(19A)-H(19F)	0.9800
C(20)-C(25)	1.396(7)
C(20)-C(21)	1.398(7)
C(21)-C(22)	1.386(9)
C(21)-H(21A)	0.9500
C(22)-C(23)	1.350(9)
C(22)-H(22A)	0.9500
C(23)-C(24)	1.394(6)
C(23)-H(23A)	0.9500
C(24)-C(25)	1.405(6)
C(24)-C(26)	1.525(7)
C(26)-C(27)	1.522(8)
C(26)-C(28)	1.526(7)
C(26)-H(26A)	1.0000
C(27)-H(27A)	0.9800
C(27)-H(27B)	0.9800

C(27)-H(27C)	0.9800
C(28)-H(28A)	0.9800
C(28)-H(28B)	0.9800
C(28)-H(28C)	0.9800
C(29)-C(30)	1.505(8)
C(29)-H(29A)	0.9900
C(29)-H(29B)	0.9900
C(30)-C(31)	1.24(3)
C(30)-H(30A)	0.9900
C(30)-H(30B)	0.9900
C(31A)-C(32)	1.511(10)
C(31A)-H(31A)	0.9900
C(31A)-H(31B)	0.9900
C(31)-C(32)	1.50(3)
C(31)-H(31C)	0.9900
C(31)-H(31D)	0.9900
C(33)-C(34)	1.406(14)
C(33)-H(33A)	0.9900
C(33)-H(33B)	0.9900
C(34)-C(35)	1.452(9)
C(34)-H(34A)	0.9900
C(34)-H(34B)	0.9900
C(35)-C(35)#1	1.51(3)
C(35)-H(35A)	0.9600
C(35)-H(35B)	0.9599
Sb(1)-F(6)	1.821(6)
Sb(1)-F(5)	1.823(7)
Sb(1)-F(4)	1.845(6)
Sb(1)-F(2)	1.846(4)
Sb(1)-F(1)	1.860(4)
Sb(1)-F(3)	1.863(6)
C(36)-Cl(2)	1.711(14)
C(36)-Cl(1)	1.751(13)
C(36)-H(36A)	0.9900
C(36)-H(36B)	0.9900
N(1)-Pd(1)-C(29)	97.07(17)
N(1)-Pd(1)-O(1)	171.03(13)
C(29)-Pd(1)-O(1)	91.76(17)
N(1)-Pd(1)-N(2)	77.60(13)
C(29)-Pd(1)-N(2)	174.56(17)
O(1)-Pd(1)-N(2)	93.59(14)
C(32)-O(1)-Pd(1)	130.1(3)
C(32)-O(2)-C(33)	117.9(6)
C(14)-N(1)-C(25)	119.4(3)
C(14)-N(1)-Pd(1)	116.9(3)
C(25)-N(1)-Pd(1)	123.5(3)
C(13)-N(2)-C(9)	122.6(3)
C(13)-N(2)-Pd(1)	114.2(3)
C(9)-N(2)-Pd(1)	123.2(3)
C(2)-C(1)-H(1A)	109.5

C(2)-C(1)-H(1B)	109.5
H(1A)-C(1)-H(1B)	109.5
C(2)-C(1)-H(1C)	109.5
H(1A)-C(1)-H(1C)	109.5
H(1B)-C(1)-H(1C)	109.5
C(2)-C(1A)-H(1AA)	109.5
C(2)-C(1A)-H(1AB)	109.4
H(1AA)-C(1A)-H(1AB)	109.5
C(2)-C(1A)-H(1AC)	109.5
H(1AA)-C(1A)-H(1AC)	109.5
H(1AB)-C(1A)-H(1AC)	109.5
C(3)-C(2)-C(1A)	116.3(9)
C(3)-C(2)-C(4)	120.6(8)
C(1A)-C(2)-C(4)	116.3(7)
C(3)-C(2)-C(3A)	26.7(7)
C(1A)-C(2)-C(3A)	111.3(6)
C(4)-C(2)-C(3A)	106.9(6)
C(3)-C(2)-C(1)	115.6(6)
C(1A)-C(2)-C(1)	16.6(8)
C(4)-C(2)-C(1)	107.2(6)
C(3A)-C(2)-C(1)	103.1(9)
C(3)-C(2)-H(2A)	103.8
C(1A)-C(2)-H(2A)	87.7
C(4)-C(2)-H(2A)	103.7
C(3A)-C(2)-H(2A)	130.5
C(1)-C(2)-H(2A)	103.7
C(2)-C(3)-H(3A)	109.4
C(2)-C(3)-H(3B)	109.5
H(3A)-C(3)-H(3B)	109.5
C(2)-C(3)-H(3C)	109.5
H(3A)-C(3)-H(3C)	109.5
H(3B)-C(3)-H(3C)	109.5
C(2)-C(3A)-H(3AA)	109.4
C(2)-C(3A)-H(3AB)	109.5
H(3AA)-C(3A)-H(3AB)	109.5
C(2)-C(3A)-H(3AC)	109.5
H(3AA)-C(3A)-H(3AC)	109.5
H(3AB)-C(3A)-H(3AC)	109.5
C(5)-C(4)-C(9)	117.6(4)
C(5)-C(4)-C(2)	120.6(5)
C(9)-C(4)-C(2)	121.8(4)
C(6)-C(5)-C(4)	120.9(5)
C(6)-C(5)-H(5A)	119.5
C(4)-C(5)-H(5A)	119.5
C(5)-C(6)-C(7)	120.3(4)
C(5)-C(6)-H(6A)	119.8
C(7)-C(6)-H(6A)	119.9
C(6)-C(7)-C(8)	121.0(5)
C(6)-C(7)-H(7A)	119.5
C(8)-C(7)-H(7A)	119.5
C(9)-C(8)-C(7)	117.9(4)

C(9)-C(8)-C(11)	120.6(5)
C(7)-C(8)-C(11)	121.5(5)
C(8)-C(9)-C(4)	122.3(4)
C(8)-C(9)-N(2)	119.8(4)
C(4)-C(9)-N(2)	117.7(4)
C(11)-C(10)-H(10A)	109.7
C(11)-C(10)-H(10B)	109.3
H(10A)-C(10)-H(10B)	109.5
C(11)-C(10)-H(10C)	109.4
H(10A)-C(10)-H(10C)	109.5
H(10B)-C(10)-H(10C)	109.5
C(11)-C(10A)-H(10D)	109.6
C(11)-C(10A)-H(10E)	109.4
H(10D)-C(10A)-H(10E)	109.5
C(11)-C(10A)-H(10F)	109.5
H(10D)-C(10A)-H(10F)	109.5
H(10E)-C(10A)-H(10F)	109.5
C(12)-C(11)-C(10)	120.5(7)
C(12)-C(11)-C(8)	114.6(7)
C(10)-C(11)-C(8)	115.5(6)
C(12)-C(11)-C(12A)	7.5(7)
C(10)-C(11)-C(12A)	127.9(8)
C(8)-C(11)-C(12A)	108.1(7)
C(12)-C(11)-C(10A)	97.6(12)
C(10)-C(11)-C(10A)	39.2(7)
C(8)-C(11)-C(10A)	105.3(6)
C(12A)-C(11)-C(10A)	103.4(8)
C(12)-C(11)-H(11A)	100.4
C(10)-C(11)-H(11A)	100.2
C(8)-C(11)-H(11A)	100.4
C(12A)-C(11)-H(11A)	98.6
C(10A)-C(11)-H(11A)	138.6
C(11)-C(12)-H(12A)	109.5
C(11)-C(12)-H(12B)	109.4
H(12A)-C(12)-H(12B)	109.5
C(11)-C(12)-H(12C)	109.5
H(12A)-C(12)-H(12C)	109.5
H(12B)-C(12)-H(12C)	109.5
C(11)-C(12A)-H(12D)	109.5
C(11)-C(12A)-H(12E)	109.5
H(12D)-C(12A)-H(12E)	109.5
C(11)-C(12A)-H(12F)	109.5
H(12D)-C(12A)-H(12F)	109.5
H(12E)-C(12A)-H(12F)	109.5
N(2)-C(13)-C(14)	115.0(3)
N(2)-C(13)-C(15)	125.5(4)
C(14)-C(13)-C(15)	119.5(4)
N(1)-C(14)-C(16)	124.1(4)
N(1)-C(14)-C(13)	116.2(4)
C(16)-C(14)-C(13)	119.6(4)
C(13)-C(15)-H(15A)	109.5

C(13)-C(15)-H(15B)	109.5
H(15A)-C(15)-H(15B)	109.5
C(13)-C(15)-H(15C)	109.5
H(15A)-C(15)-H(15C)	109.5
H(15B)-C(15)-H(15C)	109.5
C(14)-C(16)-H(16A)	109.5
C(14)-C(16)-H(16B)	109.4
H(16A)-C(16)-H(16B)	109.5
C(14)-C(16)-H(16C)	109.5
H(16A)-C(16)-H(16C)	109.5
H(16B)-C(16)-H(16C)	109.5
C(18)-C(17)-H(17A)	109.5
C(18)-C(17)-H(17B)	109.4
H(17A)-C(17)-H(17B)	109.5
C(18)-C(17)-H(17C)	109.5
H(17A)-C(17)-H(17C)	109.5
H(17B)-C(17)-H(17C)	109.5
C(18)-C(17A)-H(17D)	109.5
C(18)-C(17A)-H(17E)	109.4
H(17D)-C(17A)-H(17E)	109.5
C(18)-C(17A)-H(17F)	109.5
H(17D)-C(17A)-H(17F)	109.5
H(17E)-C(17A)-H(17F)	109.5
C(19A)-C(18)-C(17A)	114.5(6)
C(19A)-C(18)-C(20)	113.3(6)
C(17A)-C(18)-C(20)	113.0(6)
C(19A)-C(18)-C(17)	125.4(8)
C(17A)-C(18)-C(17)	12.7(9)
C(20)-C(18)-C(17)	109.6(6)
C(19A)-C(18)-C(19)	30.5(7)
C(17A)-C(18)-C(19)	92.5(8)
C(20)-C(18)-C(19)	107.0(5)
C(17)-C(18)-C(19)	105.1(6)
C(19A)-C(18)-H(18A)	82.0
C(17A)-C(18)-H(18A)	119.1
C(20)-C(18)-H(18A)	111.6
C(17)-C(18)-H(18A)	111.6
C(19)-C(18)-H(18A)	111.6
C(18)-C(19)-H(19A)	109.5
C(18)-C(19)-H(19B)	109.5
H(19A)-C(19)-H(19B)	109.5
C(18)-C(19)-H(19C)	109.5
H(19A)-C(19)-H(19C)	109.5
H(19B)-C(19)-H(19C)	109.5
C(18)-C(19A)-H(19D)	109.4
C(18)-C(19A)-H(19E)	109.5
H(19D)-C(19A)-H(19E)	109.5
C(18)-C(19A)-H(19F)	109.4
H(19D)-C(19A)-H(19F)	109.5
H(19E)-C(19A)-H(19F)	109.5
C(25)-C(20)-C(21)	116.8(4)

C(25)-C(20)-C(18)	122.0(4)
C(21)-C(20)-C(18)	121.2(5)
C(22)-C(21)-C(20)	121.2(5)
C(22)-C(21)-H(21A)	119.4
C(20)-C(21)-H(21A)	119.4
C(23)-C(22)-C(21)	120.4(4)
C(23)-C(22)-H(22A)	119.8
C(21)-C(22)-H(22A)	119.8
C(22)-C(23)-C(24)	121.7(5)
C(22)-C(23)-H(23A)	119.1
C(24)-C(23)-H(23A)	119.1
C(23)-C(24)-C(25)	117.1(4)
C(23)-C(24)-C(26)	120.5(4)
C(25)-C(24)-C(26)	122.3(4)
C(20)-C(25)-C(24)	122.7(4)
C(20)-C(25)-N(1)	118.6(4)
C(24)-C(25)-N(1)	118.6(4)
C(27)-C(26)-C(24)	111.1(4)
C(27)-C(26)-C(28)	111.0(5)
C(24)-C(26)-C(28)	110.9(4)
C(27)-C(26)-H(26A)	107.9
C(24)-C(26)-H(26A)	107.8
C(28)-C(26)-H(26A)	107.9
C(26)-C(27)-H(27A)	109.4
C(26)-C(27)-H(27B)	109.5
H(27A)-C(27)-H(27B)	109.5
C(26)-C(27)-H(27C)	109.5
H(27A)-C(27)-H(27C)	109.5
H(27B)-C(27)-H(27C)	109.5
C(26)-C(28)-H(28A)	109.5
C(26)-C(28)-H(28B)	109.5
H(28A)-C(28)-H(28B)	109.5
C(26)-C(28)-H(28C)	109.5
H(28A)-C(28)-H(28C)	109.5
H(28B)-C(28)-H(28C)	109.5
C(30)-C(29)-Pd(1)	113.5(4)
C(30)-C(29)-H(29A)	108.9
Pd(1)-C(29)-H(29A)	108.9
C(30)-C(29)-H(29B)	108.9
Pd(1)-C(29)-H(29B)	108.9
H(29A)-C(29)-H(29B)	107.7
C(31)-C(30)-C(29)	123.6(14)
C(31)-C(30)-H(30A)	106.0
C(29)-C(30)-H(30A)	106.5
C(31)-C(30)-H(30B)	106.7
C(29)-C(30)-H(30B)	106.5
H(30A)-C(30)-H(30B)	106.5
C(32)-C(31A)-H(31A)	109.5
C(32)-C(31A)-H(31B)	109.5
H(31A)-C(31A)-H(31B)	108.1
C(30)-C(31)-C(32)	129(2)

C(30)-C(31)-H(31C)	104.8
C(32)-C(31)-H(31C)	104.8
C(30)-C(31)-H(31D)	105.5
C(32)-C(31)-H(31D)	105.4
H(31C)-C(31)-H(31D)	105.9
O(1)-C(32)-O(2)	119.1(5)
O(1)-C(32)-O(2A)	120.4(6)
O(2)-C(32)-O(2A)	27.4(5)
O(1)-C(32)-C(31)	120.6(12)
O(2)-C(32)-C(31)	120.2(12)
O(2A)-C(32)-C(31)	113.0(12)
O(1)-C(32)-C(31A)	125.7(5)
O(2)-C(32)-C(31A)	111.3(6)
O(2A)-C(32)-C(31A)	113.5(7)
C(31)-C(32)-C(31A)	18.5(12)
C(34)-C(33)-O(2)	109.5(10)
C(34)-C(33)-H(33A)	109.7
O(2)-C(33)-H(33A)	109.7
C(34)-C(33)-H(33B)	109.8
O(2)-C(33)-H(33B)	109.8
H(33A)-C(33)-H(33B)	108.2
C(35)-C(34)-C(33)	118.3(13)
C(35)-C(34)-H(34A)	108.0
C(33)-C(34)-H(34A)	107.7
C(35)-C(34)-H(34B)	107.7
C(33)-C(34)-H(34B)	107.6
H(34A)-C(34)-H(34B)	107.1
C(34)-C(35)-C(35)#1	116.4(17)
C(34)-C(35)-H(35A)	99.9
C(35)#1-C(35)-H(35A)	100.2
C(34)-C(35)-H(35B)	112.8
C(35)#1-C(35)-H(35B)	118.4
H(35A)-C(35)-H(35B)	105.5
F(6)-Sb(1)-F(5)	91.5(5)
F(6)-Sb(1)-F(4)	178.4(5)
F(5)-Sb(1)-F(4)	89.9(5)
F(6)-Sb(1)-F(2)	88.2(4)
F(5)-Sb(1)-F(2)	90.7(4)
F(4)-Sb(1)-F(2)	92.4(4)
F(6)-Sb(1)-F(1)	89.4(3)
F(5)-Sb(1)-F(1)	90.1(3)
F(4)-Sb(1)-F(1)	89.9(4)
F(2)-Sb(1)-F(1)	177.5(2)
F(6)-Sb(1)-F(3)	88.7(5)
F(5)-Sb(1)-F(3)	178.5(4)
F(4)-Sb(1)-F(3)	89.8(5)
F(2)-Sb(1)-F(3)	90.8(3)
F(1)-Sb(1)-F(3)	88.4(3)
Cl(2)-C(36)-Cl(1)	111.0(8)
Cl(2)-C(36)-H(36A)	109.5
Cl(1)-C(36)-H(36A)	109.4

Cl(2)-C(36)-H(36B)	109.4
Cl(1)-C(36)-H(36B)	109.4
H(36A)-C(36)-H(36B)	108.0

Symmetry transformations used to generate equivalent atoms:
#1 -x+1,-y-1,-z

Table S4 Anisotropic displacement parameters ($Å^2 x 10^3$)for **3b**. The anisotropic displacement factor exponent takes the form: $-2\pi^2[h^2a^{*2}U^{11} + ... + 2 h k a^* b^* U^{12}]$

	U^{11}	U^{22}	U^{33}	U^{23}	U^{13}	U^{12}
Pd(1)	33(1)	49(1)	105(1)	22(1)	23(1)	5(1)
O(1)	49(2)	60(2)	148(4)	32(2)	41(2)	16(2)
N(1)	31(2)	47(2)	87(3)	15(2)	20(2)	4(1)
N(2)	31(2)	59(2)	80(2)	18(2)	18(2)	6(1)
C(2)	67(3)	130(6)	71(3)	-18(4)	4(3)	35(4)
C(4)	40(2)	76(3)	66(3)	-4(2)	9(2)	9(2)
C(5)	38(2)	95(4)	73(3)	10(3)	5(2)	17(2)
C(6)	42(2)	86(4)	89(4)	4(3)	20(2)	20(2)
C(7)	56(3)	83(4)	75(3)	-6(3)	24(2)	11(2)
C(8)	44(2)	79(3)	64(3)	7(2)	10(2)	7(2)
C(9)	30(2)	63(2)	63(2)	14(2)	13(2)	6(2)
C(11)	72(4)	137(6)	65(3)	18(4)	2(3)	2(4)
C(13)	29(2)	60(2)	70(3)	14(2)	14(2)	0(2)
C(14)	31(2)	52(2)	84(3)	14(2)	16(2)	2(1)
C(15)	33(2)	69(3)	100(4)	19(3)	28(2)	-1(2)
C(16)	45(2)	51(2)	101(4)	13(2)	27(2)	-3(2)
C(18)	68(3)	65(3)	86(4)	3(3)	12(3)	-4(2)
C(20)	44(2)	50(2)	90(3)	13(2)	21(2)	-1(2)
C(21)	58(3)	63(3)	102(4)	5(3)	39(3)	2(2)
C(22)	35(2)	80(3)	116(5)	11(3)	34(3)	8(2)
C(23)	32(2)	70(3)	106(4)	6(3)	15(2)	-1(2)
C(24)	36(2)	51(2)	93(3)	9(2)	22(2)	-2(2)
C(25)	26(2)	46(2)	92(3)	16(2)	22(2)	1(1)
C(26)	43(2)	66(3)	96(4)	-2(3)	19(2)	-5(2)
C(27)	197(10)	86(5)	99(5)	-2(4)	68(6)	-26(6)
C(28)	91(5)	122(6)	109(5)	-18(5)	20(4)	-43(5)
C(29)	40(2)	60(3)	125(5)	28(3)	26(3)	-2(2)
C(30)	67(3)	70(3)	154(6)	30(4)	60(4)	11(3)
C(30A)	67(3)	70(3)	154(6)	30(4)	60(4)	11(3)
C(31A)	29(4)	25(4)	67(6)	0(4)	22(4)	-3(3)
C(32)	52(3)	59(3)	97(4)	14(3)	29(2)	11(2)
C(33)	89(5)	91(5)	135(6)	13(4)	36(4)	46(4)
C(33A)	89(5)	91(5)	135(6)	13(4)	36(4)	46(4)
C(34)	280(20)	160(12)	154(11)	-28(9)	108(13)	-18(12)
C(35)	320(20)	146(12)	176(14)	-48(10)	59(14)	-14(13)
Sb(1)	52(1)	65(1)	75(1)	-17(1)	9(1)	5(1)
F(1)	66(2)	88(2)	139(3)	4(2)	14(2)	21(2)
F(2)	69(2)	123(4)	169(5)	-33(3)	-29(3)	28(2)
F(3)	63(4)	51(4)	162(10)	3(4)	19(5)	-12(3)
F(4)	175(11)	118(8)	84(6)	15(5)	72(7)	39(7)
F(5)	102(7)	72(5)	128(8)	-28(5)	6(5)	-25(5)
F(6)	90(6)	135(9)	68(4)	-25(5)	35(4)	1(6)

Sb(1A)	52(1)	65(1)	75(1)	-17(1)	9(1)	5(1)
F(1A)	66(2)	88(2)	139(3)	4(2)	14(2)	21(2)
F(2A)	69(2)	123(4)	169(5)	-33(3)	-29(3)	28(2)
F(3A)	178(15)	199(17)	260(20)	145(17)	140(16)	84(13)
F(4A)	460(40)	350(40)	190(20)	-190(20)	-130(20)	280(30)
F(5A)	96(11)	82(9)	710(60)	70(20)	90(20)	-14(7)
F(6A)	149(13)	165(16)	91(7)	-36(8)	-6(7)	67(11)
C(36)	151(11)	202(14)	141(10)	-58(9)	5(8)	6(10)
Cl(1)	134(4)	220(7)	131(4)	42(4)	47(3)	69(4)
Cl(2)	350(16)	242(12)	120(4)	9(6)	37(7)	-146(11)
C(36A)	151(11)	202(14)	141(10)	-58(9)	5(8)	6(10)
Cl(1A)	460(40)	199(15)	250(18)	-79(12)	70(20)	50(20)
Cl(2A)	470(40)	660(80)	440(50)	-250(60)	-50(40)	110(50)

Table S5 Hydrogen coordinates (x 10^4) and isotropic displacement parameters ($Å^2$x 10^3) for **3b**.

	x	y	z	U(eq)
H(1A)	7197	-1295	1893	134
H(1B)	8078	-1855	2244	134
H(1C)	7248	-1482	2737	134
H(1AA)	6666	-1253	1827	134
H(1AB)	7763	-1666	2290	134
H(1AC)	6650	-1403	2674	134
H(2A)	5389	-1839	1838	109
H(3A)	4916	-2357	2600	134
H(3B)	6350	-2354	3024	134
H(3C)	5862	-2897	2454	134
H(3AA)	5719	-2950	2534	134
H(3AB)	6338	-2437	3113	134
H(3AC)	7205	-2818	2651	134
H(5A)	7953	-2885	1739	83
H(6A)	8274	-3365	671	85
H(7A)	6889	-3200	-427	84
H(10A)	2925	-2659	-620	134
H(10B)	3771	-3269	-443	134
H(10C)	3319	-3053	-1278	134
H(10D)	3718	-3386	-569	134
H(10E)	4907	-3517	-958	134
H(10F)	3640	-3231	-1424	134
H(11A)	4379	-2123	-645	111
H(12A)	5616	-2006	-1359	134
H(12B)	4595	-2460	-1822	134
H(12C)	5924	-2728	-1414	134
H(12D)	5791	-1953	-1293	134
H(12E)	4864	-2393	-1841	134
H(12F)	6139	-2667	-1372	134
H(15A)	6072	-1295	190	98
H(15B)	5578	-682	534	98
H(15C)	5020	-873	-298	98
H(16A)	2093	-293	427	96
H(16B)	3062	-327	-137	96
H(16C)	3571	-190	717	96
H(17A)	2491	61	1933	134
H(17B)	2895	-162	2769	134
H(17C)	1445	-14	2444	134
H(17D)	2359	63	2142	134
H(17E)	2733	-281	2919	134
H(17F)	1280	-151	2583	134

H(18A)	2810	-1054	1950	88
H(19A)	1453	-1659	2641	134
H(19B)	824	-1033	2868	134
H(19C)	2272	-1183	3197	134
H(19D)	2726	-1714	2438	134
H(19E)	1293	-1612	2538	134
H(19F)	2417	-1281	3087	134
H(21A)	-392	-500	1952	85
H(22A)	-2067	-542	963	89
H(23A)	-1840	-1025	-107	83
H(26A)	944	-1803	-458	81
H(27A)	989	-788	-932	184
H(27B)	-501	-820	-1229	184
H(27C)	433	-1266	-1571	184
H(28A)	-1030	-2286	-427	161
H(28B)	-800	-2204	-1255	161
H(28C)	-1733	-1744	-932	161
H(29A)	668	-2256	1406	88
H(29B)	483	-2732	731	88
H(30A)	1452	-2967	2219	111
H(30B)	308	-3304	1711	111
H(30C)	1715	-3014	2189	111
H(30D)	326	-3238	1833	111
H(31A)	1222	-3847	979	47
H(31B)	1639	-4066	1816	47
H(31C)	1293	-3908	1384	202
H(31D)	2035	-3773	2188	202
H(33A)	5219	-3785	1144	123
H(33B)	5480	-4183	1890	123
H(33C)	4992	-3814	1044	123
H(33D)	5727	-4107	1784	123
H(34A)	6249	-4688	1023	226
H(34B)	5094	-5064	1237	226
H(35A)	5151	-4391	18	254
H(35B)	3871	-4651	117	254
H(36A)	703	-4768	-702	201
H(36B)	-63	-4423	-1413	201
H(36C)	395	-4478	-1363	201
H(36D)	-634	-4603	-850	201

Table S6 Torsion angles [°] for **3b**.

N(1)-Pd(1)-O(1)-C(32)	166.6(10)
C(29)-Pd(1)-O(1)-C(32)	-3.4(7)
N(2)-Pd(1)-O(1)-C(32)	177.5(6)
C(29)-Pd(1)-N(1)-C(14)	178.8(4)
O(1)-Pd(1)-N(1)-C(14)	8.8(15)
N(2)-Pd(1)-N(1)-C(14)	-2.3(4)
C(29)-Pd(1)-N(1)-C(25)	3.6(5)
O(1)-Pd(1)-N(1)-C(25)	-166.3(11)
N(2)-Pd(1)-N(1)-C(25)	-177.5(4)
N(1)-Pd(1)-N(2)-C(13)	0.8(4)
C(29)-Pd(1)-N(2)-C(13)	13(3)
O(1)-Pd(1)-N(2)-C(13)	-177.5(4)
N(1)-Pd(1)-N(2)-C(9)	-179.4(4)
C(29)-Pd(1)-N(2)-C(9)	-168(2)
O(1)-Pd(1)-N(2)-C(9)	2.3(4)
C(3)-C(2)-C(4)-C(5)	-69.3(11)
C(1A)-C(2)-C(4)-C(5)	80.9(10)
C(3A)-C(2)-C(4)-C(5)	-44.1(9)
C(1)-C(2)-C(4)-C(5)	65.9(9)
C(3)-C(2)-C(4)-C(9)	110.7(10)
C(1A)-C(2)-C(4)-C(9)	-99.1(8)
C(3A)-C(2)-C(4)-C(9)	135.8(7)
C(1)-C(2)-C(4)-C(9)	-114.2(8)
C(9)-C(4)-C(5)-C(6)	-0.3(8)
C(2)-C(4)-C(5)-C(6)	179.7(6)
C(4)-C(5)-C(6)-C(7)	0.2(9)
C(5)-C(6)-C(7)-C(8)	-0.4(9)
C(6)-C(7)-C(8)-C(9)	0.6(8)
C(6)-C(7)-C(8)-C(11)	-175.6(6)
C(7)-C(8)-C(9)-C(4)	-0.7(7)
C(11)-C(8)-C(9)-C(4)	175.5(5)
C(7)-C(8)-C(9)-N(2)	-175.3(4)
C(11)-C(8)-C(9)-N(2)	0.9(7)
C(5)-C(4)-C(9)-C(8)	0.5(7)
C(2)-C(4)-C(9)-C(8)	-179.4(6)
C(5)-C(4)-C(9)-N(2)	175.2(5)
C(2)-C(4)-C(9)-N(2)	-4.7(7)
C(13)-N(2)-C(9)-C(8)	-86.8(6)
Pd(1)-N(2)-C(9)-C(8)	93.5(5)
C(13)-N(2)-C(9)-C(4)	98.3(6)
Pd(1)-N(2)-C(9)-C(4)	-81.4(5)
C(9)-C(8)-C(11)-C(12)	136.0(13)
C(7)-C(8)-C(11)-C(12)	-47.9(15)
C(9)-C(8)-C(11)-C(10)	-77.3(11)
C(7)-C(8)-C(11)-C(10)	98.7(10)
C(9)-C(8)-C(11)-C(12A)	132.1(11)
C(7)-C(8)-C(11)-C(12A)	-51.8(13)
C(9)-C(8)-C(11)-C(10A)	-117.9(7)

C(7)-C(8)-C(11)-C(10A)	58.2(8)
C(9)-N(2)-C(13)-C(14)	-179.1(4)
Pd(1)-N(2)-C(13)-C(14)	0.7(6)
C(9)-N(2)-C(13)-C(15)	2.3(8)
Pd(1)-N(2)-C(13)-C(15)	-177.9(4)
C(25)-N(1)-C(14)-C(16)	0.1(8)
Pd(1)-N(1)-C(14)-C(16)	-175.3(4)
C(25)-N(1)-C(14)-C(13)	178.8(4)
Pd(1)-N(1)-C(14)-C(13)	3.4(6)
N(2)-C(13)-C(14)-N(1)	-2.7(7)
C(15)-C(13)-C(14)-N(1)	176.0(5)
N(2)-C(13)-C(14)-C(16)	176.1(5)
C(15)-C(13)-C(14)-C(16)	-5.3(8)
C(19A)-C(18)-C(20)-C(25)	-89.1(8)
C(17A)-C(18)-C(20)-C(25)	138.7(8)
C(17)-C(18)-C(20)-C(25)	125.5(8)
C(19)-C(18)-C(20)-C(25)	-121.0(7)
C(19A)-C(18)-C(20)-C(21)	89.1(9)
C(17A)-C(18)-C(20)-C(21)	-43.1(9)
C(17)-C(18)-C(20)-C(21)	-56.3(9)
C(19)-C(18)-C(20)-C(21)	57.2(8)
C(25)-C(20)-C(21)-C(22)	-1.9(7)
C(18)-C(20)-C(21)-C(22)	179.9(5)
C(20)-C(21)-C(22)-C(23)	3.0(8)
C(21)-C(22)-C(23)-C(24)	-1.3(8)
C(22)-C(23)-C(24)-C(25)	-1.5(7)
C(22)-C(23)-C(24)-C(26)	-178.3(5)
C(21)-C(20)-C(25)-C(24)	-1.1(6)
C(18)-C(20)-C(25)-C(24)	177.2(4)
C(21)-C(20)-C(25)-N(1)	-177.6(4)
C(18)-C(20)-C(25)-N(1)	0.6(6)
C(23)-C(24)-C(25)-C(20)	2.7(6)
C(26)-C(24)-C(25)-C(20)	179.4(4)
C(23)-C(24)-C(25)-N(1)	179.3(4)
C(26)-C(24)-C(25)-N(1)	-4.1(6)
C(14)-N(1)-C(25)-C(20)	-84.3(6)
Pd(1)-N(1)-C(25)-C(20)	90.7(4)
C(14)-N(1)-C(25)-C(24)	99.0(5)
Pd(1)-N(1)-C(25)-C(24)	-86.0(5)
C(23)-C(24)-C(26)-C(27)	77.7(6)
C(25)-C(24)-C(26)-C(27)	-98.8(7)
C(23)-C(24)-C(26)-C(28)	-46.3(7)
C(25)-C(24)-C(26)-C(28)	137.2(5)
N(1)-Pd(1)-C(29)-C(30)	-153.3(5)
O(1)-Pd(1)-C(29)-C(30)	25.2(6)
N(2)-Pd(1)-C(29)-C(30)	-165(2)
Pd(1)-C(29)-C(30)-C(31)	-43.0(19)
C(29)-C(30)-C(31)-C(32)	32(4)
Pd(1)-O(1)-C(32)-O(2)	168.0(6)
Pd(1)-O(1)-C(32)-O(2A)	-160.3(8)
Pd(1)-O(1)-C(32)-C(31)	-9.4(16)

Pd(1)-O(1)-C(32)-C(31A)	11.9(12)
C(33)-O(2)-C(32)-O(1)	24.2(12)
C(33)-O(2)-C(32)-O(2A)	-76.4(14)
C(33)-O(2)-C(32)-C(31)	-158.4(15)
C(33)-O(2)-C(32)-C(31A)	-176.5(9)
C(30)-C(31)-C(32)-O(1)	-2(3)
C(30)-C(31)-C(32)-O(2)	-179(2)
C(30)-C(31)-C(32)-O(2A)	151(3)
C(30)-C(31)-C(32)-C(31A)	-113(6)
C(32)-O(2)-C(33)-C(34)	-148.2(9)
O(2)-C(33)-C(34)-C(35)	62.5(17)
C(33)-C(34)-C(35)-C(35)#1	173.5(18)
